# GALNT14 promotes lung-specific breast cancer metastasis by modulating self-renewal and interaction with the lung microenvironment

**DOI:** 10.1038/ncomms13796

**Published:** 2016-12-16

**Authors:** Ki-Hoon Song, Mi So Park, Tulip S. Nandu, Shrikanth Gadad, Sang-Cheol Kim, Mi-Young Kim

**Affiliations:** 1 Department of Biological Sciences, Korea Advanced Institute of Science and Technology (KAIST), Daejon 305-701, Korea; 2 Cecil H. and Ida Green Center for Reproductive Biology Sciences and Division of Basic Reproductive Biology Research, Department of Obstetrics and Gynecology, University of Texas Southwestern Medical Center, Dallas, Texas 75390, USA; 3 Department of Biomedical Informatics, Center for Genome Science, National Institute of Health, KCDC, Choongchung-Buk-do 363-951, Korea; 4 KAIST Institute for the BioCentury, Cancer Metastasis Control Center, 291 Daehak-ro, Yuseong-gu, Daejeon 305-701, Korea

## Abstract

Some polypeptide N-acetyl-galactosaminyltransferases (GALNTs) are associated with cancer, but their function in organ-specific metastasis remains unclear. Here, we report that GALNT14 promotes breast cancer metastasis to the lung by enhancing the initiation of metastatic colonies as well as their subsequent growth into overt metastases. Our results suggest that GALNT14 augments the self-renewal properties of breast cancer cells (BCCs). Furthermore, GALNT14 overcomes the inhibitory effect of lung-derived bone morphogenetic proteins (BMPs) on self-renewal and therefore facilitates metastasis initiation within the lung microenvironment. In addition, GALNT14 supports continuous growth of BCCs in the lung by not only inducing macrophage infiltration but also exploiting macrophage-derived fibroblast growth factors (FGFs). Finally, we identify KRAS-PI3K-c-JUN signalling as an upstream pathway that accounts for the elevated expression of *GALNT14* in lung-metastatic BCCs. Collectively, our findings uncover an unprecedented role for GALNT14 in the pulmonary metastasis of breast cancer and elucidate the underlying molecular mechanisms.

## Abstract (web summary)

Polypeptide N-acetyl-galactosaminyltransferases (GALNTs) are associated with cancer, but their function in organ-specific metastasis is unclear. Here the authors show that GALNT14 promotes breast cancer metastasis to the lung by enhancing the initiation of metastatic colonies and subsequent growth.

Breast cancer metastasizes to many organs, including the lung, bones and brain, each of which imposes different requirements on incoming cancer cells for their survival and subsequent outgrowth into overt metastases^1^,^2^. Thus, organ-tropic metastatic cells must possess the following abilities: (1) to initiate metastatic colonies against anti-metastatic signals produced by the destination organ and (2) to exploit the newly encountered microenvironment for the establishment of clinical metastases^3^,^4^. The acquisition of organ-specific metastatic potential by breast cancer cells (BCCs) is generally achieved by specific sets of genes that can modulate the intrinsic cellular functions of cancer cells themselves and/or their crosstalk with stromal components^5^,^6^,^7^,^8^.

O-glycosylation, the attachment of monosaccharides to Ser and Thr residues on acceptor proteins, is one of the most common post-translational modifications and regulates various biological processes, including cell growth, signalling, protein stability and trafficking, and cell adhesion^9^,^10^,^11^. O-linked N-acetyl galactosamine (GalNAc) glycosylation (referred to as O-GalNAcylation) is one class of O-glycosylation that is initiated by the transfer of GalNAc from UDP-GalNAc to acceptor proteins by a large family of enzymes, called polypeptide N-acetyl galactosaminyl transferases (GALNTs)^12^,^13^. To date, 20 GALNT family members have been identified in humans, and these isozymes have been shown to exhibit differential but overlapping substrate specificities and cell type-dependent expression patterns^14^,^15^.

In addition to their roles in normal cellular processes, the altered expression of *GALNTs,* accompanied by changes in O-glycan compositions, has been found in several disease states, including cancer^9^,^10^,^16^. However, the functional roles of GALNTs identified to date in cancer are mostly limited to their involvement in cancer cell motility or growth^15^,^17^,^18^,^19^,^20^,^21^. Furthermore, the potential function of GALNTs on cancer progression, especially in site-specific metastasis, is poorly understood.

Thus, we set out to identify the GALNT(s) that promote(s) the organ-specific metastasis of breast cancer and to investigate the underlying mechanisms. Our study reveals that GALNT14 specifically promotes breast cancer metastasis to the lung, by accelerating the initiation of metastatic colonies as well as their subsequent growth into macrometastases. Specifically, we show that GALNT14 enhances the aforementioned processes by enabling BCCs to (1) overcome the inhibitory effect of lung-derived bone morphogenetic proteins (BMPs) on self-renewal, (2) create a favourable microenvironment in the lung and (3) exploit growth signals produced by stromal cells in the lung. Furthermore, we provide molecular insights on how GALNT14 orchestrates these processes.

## Results

### *GALNT14* expression is selectively linked to lung metastasis

**Figure 1:**
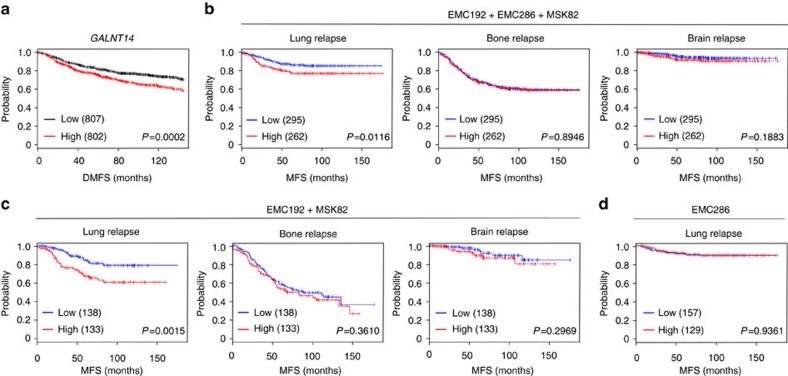
*GALNT14* expression is specifically associated with breast cancer relapse to the lung. (**a**) Kaplan–Meier plot of distant metastasis-free survival (DMFS) of breast cancer patients, stratified by expression of *GALNT14* in their primary tumours. (**b**–**d**) Lung, bone or brain metastasis-free survival (MFS) in the combined cohort of EMC192, EMC286 and MSK82 (**b**) or EMC192 and MSK82 (**c**), based on the expression of *GALNT14* in primary tumours. Kaplan–Meier analysis of lung MFS in the EMC286 cohort (**d**). *P* values were calculated using a log rank test.

To identify a GALNT(s) that contribute(s) to the breast cancer metastasis, we first searched for the *GALNT* family member(s) whose expression in primary breast tumours correlated with a higher risk of distant metastasis. Kaplan–Meier analysis of publically available microarray data^22^ revealed that only *GALNT14* was strongly associated with distant metastasis-free survival (DMFS) (Fig. 1a and Supplementary Fig. 1a).

To further assess the prognostic value of *GALNT14* in breast cancer, the association between *GALNT14* expression and organ-specific metastasis was analysed in a combined microarray data set (EMC192, MSK82 and EMC286)^5^,^8^. Interestingly, primary tumours with high *GALNT14* expression exhibited a significant association with decreased lung metastasis-free survival (MFS), but not with brain or bone MFS (Fig. 1b). Furthermore, this association was still observed in the combined EMC192/MSK82 data set, which only consists of advanced breast cancers (Fig. 1c and Supplementary Table 1). When individually analysed, *GALNT14* exhibited a statistically significant association with lung MFS in the EMC192 cohort. In the MSK82 data set, *GALNT14* showed a clear tendency to lung metastasis-specific association (Supplementary Fig. 1b). In contrast to advanced breast cancers, *GALNT14* expression levels in early-stage tumours (EMC286)^5^ had no association with lung MFS (Fig. 1d and Supplementary Fig. 1c).

Taken together, our data indicate that *GALNT14* expression correlates with lung metastasis in patients with advanced breast cancer.

### GALNT14 enhances the lung colonization ability of BCCs

**Figure 2:**
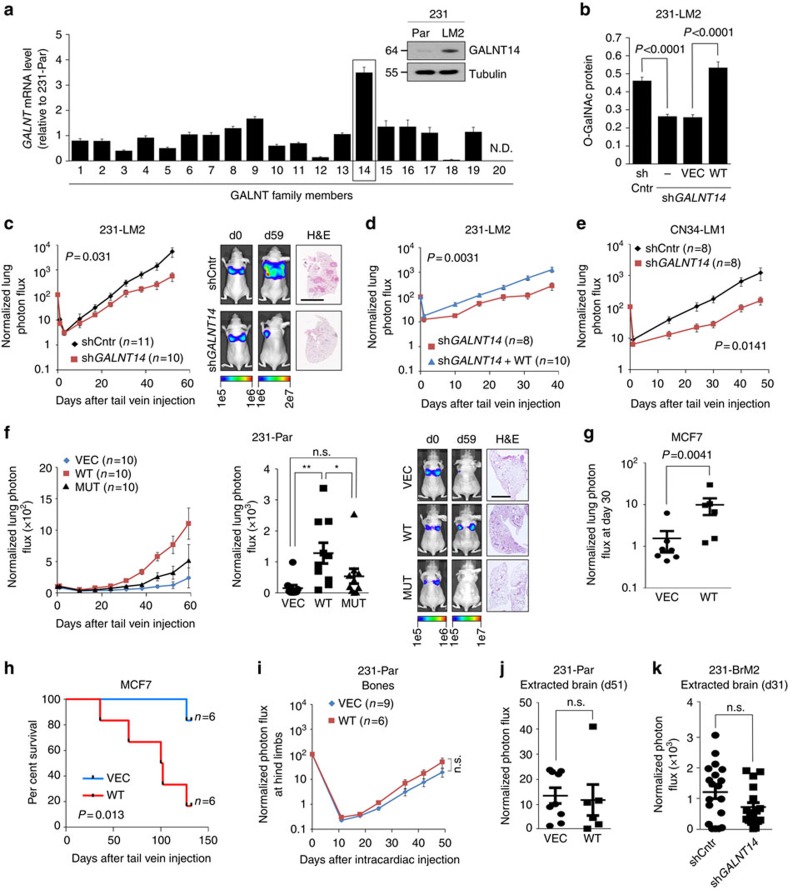
GALNT14 selectively promotes lung metastasis. (**a**) Fold changes in the expression of *GALNT1* through *GALNT20* in 231-LM2 relative to 231-Par. N.D.: Not detected. Relative GALNT14 protein levels in these cell lines are also shown. (**b**) Comparison of O-GalNAcylation in control (shCntr), *GALNT14-*silenced (sh*GALNT14)* and rescued (WT) 231-LM2 cells. Cell lysates were subjected to Vicia villosa lectin (VVA)-ELISA (VEC: control vector, WT: wild-type *GALNT14*. *n=*6. *P* values, two-tailed unpaired Student's *t*-test. (**c**) The indicated 231-LM2 cells were introduced intravenously into immunocompromised mice. Lung colonization was analysed by BLI. Left: Bioluminescent signals from the lungs normalized to signals at day 0. Right: Representative bioluminescent images of mice and histological staining (H&E) of lung sections. Scale bar, 5 mm. (**d**) Comparative lung colonization abilities of *GALNT14*-silenced and rescued 231-LM2 cells. (**e**) Experimental lung metastasis assays with the indicated CN34-LM1 cells. (**f**) Experimental lung metastasis assays with 231-Par cells expressing control vector (VEC), wild-type (WT) or catalytic mutant (MUT) *GALNT14*. Left: Normalized lung BLI signals. Middle: a scatter plot of normalized lung BLI signals at day 59. Right: Representative images of mice and histological staining (H&E) of lung sections. Scale bar, 5 mm. (**g**,**h**) Relative lung colonization by MCF7 cells expressing control vector (VEC) or wild-type *GALNT14* (WT) (**g**). Kaplan–Meier survival curves of mice injected with these cells are shown in (**h**). *P* values in **h** were calculated using a log rank test. (**i**,**j**) The indicated 231-Par cells were inoculated into mice via intracardiac injection. Normalized BLI signals in the hind limbs (**i**) and extracted brains at day 51 post the injection (**j**) are shown. *n*=9 (VEC) and 6 (WT). (**k**) Comparison of brain-metastatic activities of the indicated 231-BrM2 cells. Normalized BLI signals in extracted brains at day 31 after intracardiac injection are shown. *n=*19 (shCntr) and 17 (sh*GALNT14*). *P* values: one-tailed Mann–Whitney test unless indicated otherwise. *P<0.05; **P<0.001; n.s. *P*>0.05. Data are mean±s.e.m. qRT-PCR data shown in **a** is a representative of two independent experiments, each with triplicate samples. qRT-PCR, quantitative real-time PCR.

Given the clinical evidence suggesting a potential role of GALNT14 in pulmonary metastasis, we sought to experimentally evaluate this possibility. We first analysed the expression of all 20 GALNT family members in the parental MDA-MB-231 BCC line (hereafter 231-Par) and 231-LM2, a highly lung-metastatic subline derived from 231-Par (ref. 23). In accordance with clinical data analysis, *GALNT14* expression showed the greatest increase in 231-LM2 cells (Fig. 2a). Similarly, lung-metastatic mouse mammary tumour cell lines exhibited increased *GALNT14* expression compared with those with no detectable lung-metastatic potential (Supplementary Fig. 2a)^24^.

Next, we investigated whether GALNT14 is required for lung colonization by BCCs. shRNA-mediated knockdown of *GALNT14* markedly decreased overall O-GalNAcylation in 231-LM2 cells, which was restored in the *GALNT14*-rescued cells (Fig. 2b and Supplementary Fig. 2b). This finding suggests that GALNT14 is one of the major contributors to O-GalNAcylation in these cells. More importantly, *GALNT14* knockdown considerably attenuated lung colonization by 231-LM2 cells (Fig. 2c), whereas restoring its expression recovered lung-metastatic activity (Fig. 2d). Similarly, *GALNT14* knockdown in CN34-LM1, a lung-metastatic derivative of another triple-negative BCC line CN34 (ref. 25), reduced lung metastasis (Fig. 2e and Supplementary Fig. 2c). Conversely, the ectopic expression of GALNT14 in 231-Par cells enhanced overall O-GalNAcylation and lung colonization in an enzymatic activity-dependent manner^26^ (Fig. 2f and Supplementary Fig. 2d,e) without causing significant changes in their general growth in the primary site (Supplementary Fig. 2f). Furthermore, GALNT14 overexpression in the very weakly metastatic MCF7 cell line increased their pulmonary metastatic potential, resulting in shorter overall survival (Fig. 2g,h and Supplementary Fig. 2g). Thus, our data strongly suggest that GALNT14 plays a critical role in lung colonization by BCCs.

Our clinical data analysis indicated that GALNT14 may promote lung-specific metastasis of BCCs (Fig. 1b,c). Thus, we examined whether GALNT14 contributes to breast cancer metastasis to other organs, including the bones and brain. In contrast to a considerable increase in lung metastasis by GALNT14-overexpressing 231-Par cells (Fig. 2f), no significant changes were observed in the formation of bone or brain metastases (Fig. 2i,j). Further supporting this observation, the silencing of *GALNT14* in 231-BrM2 cells (Supplementary Fig. 2h), a brain-metastatic subline of 231-Par, had no effect on their brain-metastatic potential (Fig. 2k).

Collectively, our results suggest that GALNT14 selectively potentiates breast cancer metastasis to the lung.

### GALNT14 confers self-renewal capability in BCCs

To delineate the cellular mechanisms by which GALNT14 promotes pulmonary metastasis, we first examined whether GALNT14 affects the following intrinsic properties of BCCs that are generally associated with increased metastatic potential: (1) general growth rate *in vitro*; (2) resistance to detachment-induced programmed cell death, that is, anoikis; (3) adhesion to extracellular matrix and endothelial cells; (4) invasion and migration; and (5) epithelial-mesenchymal transition. *GALNT14* knockdown had no effect on any of these characteristics (Supplementary Fig. 3a–g), which indicates that GALNT14 promotes lung metastasis by mechanisms distinct from the aforementioned processes.

**Figure 3:**
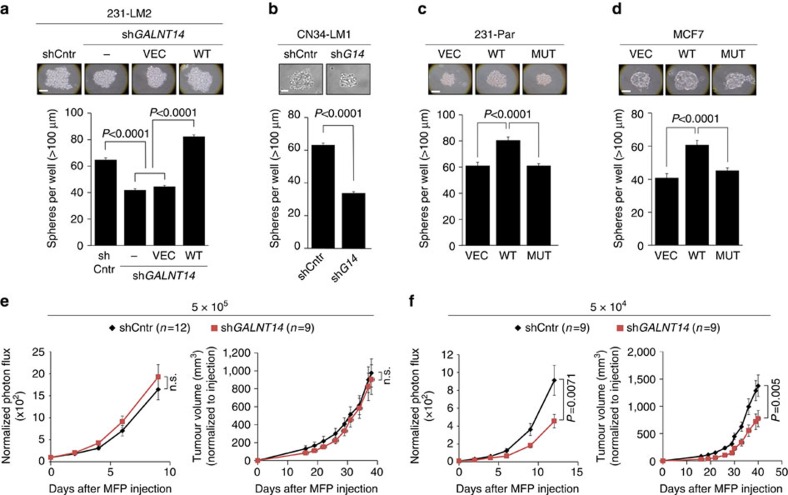
GALNT14 confers self-renewal capability on BCCs. (**a**,**b**) Comparative sphere-forming abilities of the indicated 231-LM2 (**a**) and CN34-LM1 (**b**) cells. Single cell populations were plated into ultra-low attachment plates and the number of spheres over 100 μm was counted after 7 days. Representative images (top) and the total number of spheres (bottom) are shown. Scale bar, 50 μm. *n=*6. (**c**,**d**) Same as in **b**, but 231-Par (**c**) or MCF7 (**d**) cells expressing control vector (VEC), wild-type (WT) or mutant (MUT) *GALNT14* were used. Scale bar, 50 μm. *n=*6. (**e**) Normalized photon flux from MFPs inoculated with 231-LM2 cells expressing shCntr or sh*GALNT14* (left) and their comparative tumour growth rates (right) upon injection of 5 × 10^5^ cells. (**f**) Similar experiments as in **e** except 5 × 10^4^ cells were injected. *P* values in **a**–**d** were calculated using two-tailed unpaired Student's *t*-test and **e**,**f** using one-tailed Mann–Whitney test. Data are mean±s.e.m.

Based on these data, we then considered a potential role of GALNT14 in the later stages of the metastatic process, such as the initiation of metastatic colonies and their subsequent outgrowth in the lung parenchyma. To this end, we first examined whether GALNT14 confers BCCs with self-renewal ability, which is often associated with the metastasis-initiating potential of BCCs^27^,^28^. Interestingly, *GALNT14* knockdown in 231-LM2 and CN34-LM1 significantly impaired sphere formation (Fig. 3a,b). Likewise, the ectopic expression of the wild-type GALNT14, but not the catalytic mutant, increased the sphere-forming capabilities of 231-Par and MCF7 cells (Fig. 3c,d). Thus, our findings indicate that GALNT14 may endow BCCs with lung-metastatic potential by enhancing self-renewal abilities.

### GALNT14 promotes mammary tumour initiation

It has been suggested that the self-renewal abilities of BCCs are closely associated with their tumour-initiating abilities *in vivo*^29^. Thus, the enhanced self-renewal of BCCs conferred by GALNT14 led us to examine the role of this enzyme during the initial stage of mammary tumour formation. To this end, we inoculated mouse mammary fat pads (MFPs) with control or *GALNT14*-silenced 231-LM2 cells at two different concentrations (5 × 10^5^ or 5 × 10^4^) and monitored early tumourigenesis by measuring bioluminescent signals emitted from MFPs. Interestingly, *GALNT14* knockdown attenuated the initial growth of mammary tumours only when 5 × 10^4^ cells were injected, which led to decreased tumour volume at the later stage (Fig. 3e,f). This finding is in accordance with previous studies showing that the differences in tumour-initiating abilities of BCCs are generally observed only when limited numbers of cells are implanted^30^,^31^. Collectively, our data suggest that GALNT14 not only promotes lung metastasis but also mammary tumour initiation. This is in accordance with previous studies showing that the genes that promote organ-specific metastasis can also contribute to primary tumour formation^6^,^23^,^30^,^32^.

### GALNT14 enhances BCC self-renewal by suppressing BMP signalling

**Figure 4:**
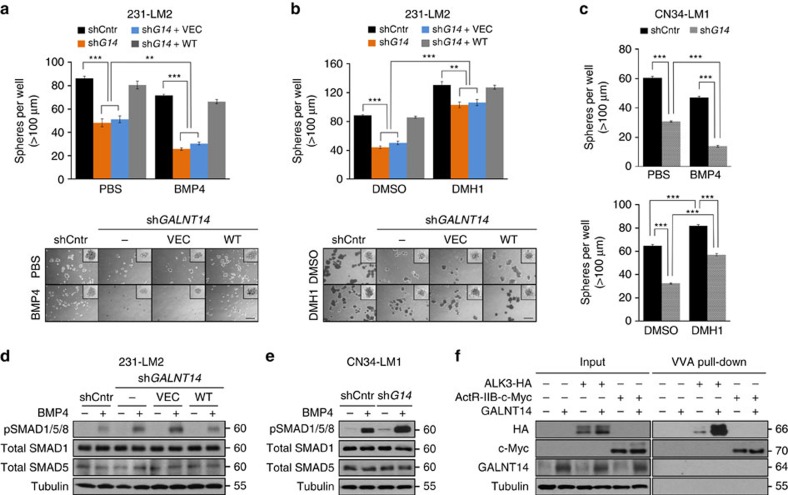
GALNT14 promotes self-renewal of BCCs by suppressing BMP signalling. (**a**) The role of GALNT14 in overcoming inhibitory effects of BMPs on self-renewal. Top: The number of spheres formed by the indicated 231-LM2 cells in the presence of PBS or BMP4 (30 ng ml^−1^). Bottom: Representative images of spheres. Images of single sphere are shown in the inset. sh*G14* indicates sh*GALNT14*. Scale bar, 500 μm*. n=*6. (**b**) Similar experiment as in **a** except that cells were treated with DMSO or BMPR kinase inhibitor DMH1 (5 μM). Scale bar, 500 μm. *n*=6. (**c**) Similar experiment as in **a**,**b** except that control (shCntr) and *GALNT14*-silenced (sh*G14*) CN34-LM1 cells were used. BMP4 (10 ng ml^−1^). *n=*6 (**d**,**e**) The indicated 231-LM2 cells (**d**) and CN34-LM1 cells (**e**) were treated with PBS or BMP4 (30 ng ml^−1^ for 231-LM2, 10 ng ml^−1^ for CN34-LM1), followed by immunoblotting analyses with antibodies against phospho-SMAD1/5/8, total SMAD1, and SMAD5. (**f**) O-GalNAcylation of BMPRs. HEK 293T cells were transfected with BMPR1A (ALK3), ActR-IIB and GALNT14 expression vectors as indicated and subjected to pull-down with Vicia villosa lectin (VVA)-agarose, followed by the western blot analyses with the indicated antibodies. *P* values were calculated using two-tailed unpaired Student's *t*-test. **P<0.001; ***P<0.0001. Data in **a**–**c** are mean±s.e.m. from three independent experiments and western blots in **d**–**f** are representatives of two to three independent experiments.

Lung-metastatic BCCs must be able to initiate metastatic colonies against anti-metastatic signals produced by the lung. Since our results implied the role for GALNT14 in promotion of self-renewal and lung-specific metastasis, we investigated whether GALNT14 enables BCCs to overcome the inhibitory effect of lung-derived anti-metastatic signals on self-renewal. To this end, we analysed the sphere-forming abilities of control and *GALNT14*-knockdown 231-LM2 cells in the presence of BMPs because BMPs are highly present in the lung compared with other organs and they are known to prevent the self-renewal of incoming BCCs in the lung parenchyma^30^. Interestingly, *GALNT14*-silenced 231-LM2 cells exhibited increased susceptibility to the BMP-mediated suppression of sphere formation (Fig. 4a). In addition, inhibiting BMP signalling by DMH1, the BMP receptor (BMPR) kinase inhibitor, greatly increased sphere formation by *GALNT14*-silenced 231-LM2 (Fig. 4b) while causing only a minimal increase with control 231-LM2 cells. Similar results were observed with CN34-LM1 (Fig. 4c). Thus, our data support that GALNT14 allows BCCs to overcome the inhibitory effect of BMPs on BCC self-renewal.

To further delineate the mechanism by which GALNT14 enables BCCs to resist the suppressive effect of BMPs on self-renewal, we analysed downstream BMP signalling. The BMP signalling cascade is initiated by the binding of BMP ligands to heteromeric type I-type II receptor complexes, which subsequently induces the phosphorylation of SMAD1/5/8 and their interaction with SMAD4. These proteins then translocate to the nucleus, where they regulate the expression of their target genes^33^. Notably, *GALNT14* knockdown in 231-LM2 and CN34-LM1 cells led to a greater increase in the BMP-induced phosphorylation of SMAD1/5/8 compared with the control cells (Fig. 4d,e), and this phosphorylation was accompanied by increased interactions between pSMAD1/5/8 and SMAD4 (Supplementary Fig. 4a). Thus, our data indicate that GALNT14 suppresses BMP signalling and therefore, promotes BCC self-renewal within the BMP-rich lung microenvironment.

The BMP pathway is closely related to the TGFβ pathway, and they often operate antagonistically^34^. Thus, we examined whether GALNT14 exerts its self-renewal promoting effect on BCCs by orchestrating crosstalk between these two pathways. In contrast to the significant increase in BMP-induced phosphorylation of SMAD1/5/8 upon *GALNT14* knockdown (Fig. 4d), no changes in TGFβ-induced SMAD2/3 phosphorylation, an indicator of activated TGFβ signalling, was observed (Supplementary Fig. 4b,c). Furthermore, *GALNT14* knockdown had no effect on the interaction between pSMAD2/3 and SMAD4 (Supplementary Fig. 4d). Therefore, these data support that GALNT14-induced self-renewal is most likely due to direct suppression of the BMP signalling by GALNT14 rather than the activation of the TGFβ pathway.

The O-GalNAcylation of cell-surface receptors by GALNTs has been shown to regulate downstream signalling cascades^20^,^35^,^36^. Since our data suggest that GALNT14 inhibits BMP signalling, we examined whether GALNT14 exerts this effect by the O-GalNAcylation of BMPRs. To this end, we transiently expressed the BMPR 1A (ALK3) or Activin receptor IIB (ActR-IIB), one of the type II receptors, and performed pull-down assays with VVA (vicia villosa lectin), which specifically binds to GalNAc residues on modified proteins. We observed a marked increase in O-GalNAcylation of ALK3, but not ActR-IIB, upon the co-expression of GALNT14 (Fig. 4f), and this increase was ablated by benzyl-GalNAc, a commonly used O-GalNAcylation inhibitor (Supplementary Fig. 5a). Furthermore, the O-GalNAcylation of endogenous ALK3 was detected in 231-LM2 cells, which was abrogated upon *GALNT14* knockdown (Supplementary Fig. 5b). Our data suggest that the GALNT14-mediated O-GalNAcylation of ALK3 may impair the BMP responsiveness of BCCs, which subsequently enhances their self-renewal.

### SOX4 is a key mediator of GALNT14 in lung metastasis

**Figure 5:**
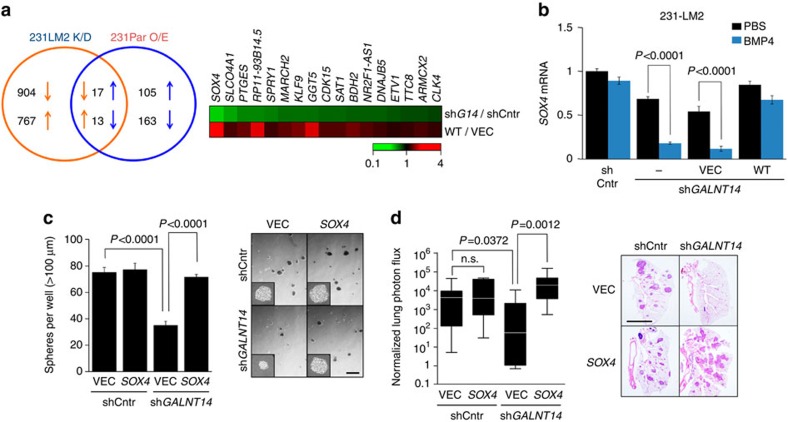
GALNT14 promotes lung metastasis initiation by reversing the BMP-mediated suppression of *SOX4* expression. (**a**) Left: A Venn diagram representing the number of differentially expressed genes (*P*<0.05) upon knockdown and overexpression of *GALNT14* in 231-LM2 (231-LM2 K/D) and in 231-Par (231-Par O/E), respectively. Right: Heat-map representation of 17 genes whose expression was decreased and increased upon knockdown of *GALNT14* in 231-LM2 and overexpression in 231-Par, respectively. (**b**) Comparison of *SOX4* transcript levels in the indicated 231-LM2 cells upon treatment with PBS or BMP4. *n*=6. (**c**) Recovery of sphere formation by *GALNT14-*silenced 231-LM2 cells upon restoration of *SOX4* expression. Left: Comparison of sphere-forming abilities among the indicated 231-LM2 cells (VEC: control vector, *SOX4*: *SOX4* expression vector). Right: Representative sphere images. Scale bar, 500 μm. *n*=6. (**d**) Lung colonization by cells used in **c**. *n*=9 (shCntr+VEC), 8 (shCntr+*SOX4*), 8 (sh*GALNT14*+VEC) and 9 (sh*GALNT14*+*SOX4*). A mouse with an unusually high-normalized photon flux of 810,000 in the sh*GALNT14*-VEC cohort was excluded. Representative H&E staining of lung sections are shown on the right. Scale bar, 5 mm. *P* values based on Mann–Whitney test. Boxes: 25–75 percentiles, whiskers: 5 and 95 percentiles, line: median. *P* values were calculated using two-tailed unpaired Student's *t*-test unless indicated otherwise. Data are mean±s.e.m. unless indicated otherwise.

Having uncovered the role of GALNT14 in promoting BCC self-renewal, we then sought to identify a downstream effector of GALNT14 in this process. To this end, we searched for genes whose expression was regulated by GALNT14 by performing a genome-wide RNA-seq analysis. *GALNT14* knockdown in 231-LM2 led to 780 up- and 921 downregulated genes with a p value smaller than 0.05, while its ectopic expression in 231-Par cells resulted in 122 up- and 176 downregulated genes. The overlap between these two data sets yielded a list containing 17 up- and 13 downregulated genes by GALNT14 (Fig. 5a, Venn diagram).

Among the 17 upregulated genes (Fig. 5a heat map and Supplementary Table 2), the progenitor transcription factor Sex-Determining Region Y-box4 (SOX4) caught our attention for the following reasons: (1) its expression exhibited the greatest fold-change upon both knockdown and overexpression of *GALNT14*; (2) SOX4 has been shown to be regulated by BMP signalling, which was identified as a target pathway of GALNT14 in the current study; and (3) increased *SOX4* expression in glioma-initiating cells has been reported^37^. In breast cancer, *SOX4* is associated with cancer progression^25^,^38^,^39^,^40^, yet its role during the metastasis initiation step remains unclear. Furthermore, the contribution of GALNTs to pulmonary metastasis through the regulation of *SOX4* is unknown.

Quantitative real-time PCR (qRT-PCR) analysis confirmed that GALNT14 indeed upregulates *SOX4* expression in 231 and CN34 cells (Supplementary Fig. 6a–c). More importantly, BMP treatment markedly decreased *SOX4* transcripts in *GALNT14*-silenced 231-LM2 cells while causing no significant changes in control and rescued cells (Fig. 5b). This suggests that GALNT14 increases *SOX4* transcription in lung-metastatic BCCs by suppressing BMP signalling. Further supporting this, 231-LM2 cells, which express elevated *GALNT14* (Fig. 2a) and *SOX4* (ref. 25) compared with 231-Par cells, exhibited attenuated BMP responsiveness as well as no changes in *SOX4* expression upon BMP4 treatment. In contrast, BMP treatment markedly reduced *SOX4* transcripts in 231-Par cells (Supplementary Fig. 6d).

Based on our findings indicating a potential link among GALNT14, BMP and SOX4, we examined whether SOX4 functions as a key mediator of GALNT14 in BCC self-renewal. The re-expression of *SOX4* in *GALNT14*-silenced 231-LM2 recovered their sphere-forming activity to a level similar to control cells without affecting the general growth rate, while *SOX4* overexpression in control 231-LM2 caused no further increase in sphere formation (Fig. 5c and Supplementary Fig. 6e,f). Interestingly, reduced *SOX4* level upon *GALNT14* knockdown also led to decreased expression of the embryonic stem cell markers *SOX2* and *OCT4* (Supplementary Fig. 6g,h) and their expression levels were restored in *SOX4*-rescued 231-LM2 cells (Supplementary Fig. 6i). Thus, these data indicate that SOX4 is the main mediator of GALNT14 in BCC self-renewal, potentially by regulating *SOX2* and *OCT4* expression. Finally, the re-expression of *SOX4* in *GALNT14*-silenced 231-LM2 cells recovered their lung-metastatic potential (Fig. 5d).

Collectively, our findings suggest that GALNT14 upregulates *SOX4* expression by suppressing BMP signalling in lung-metastatic BCCs, which then enhances BCC self-renewal and thus, the initiation of metastatic colonies in the lung parenchyma.

### GALNT14 enables BCCs to exploit the lung microenvironment

**Figure 6:**
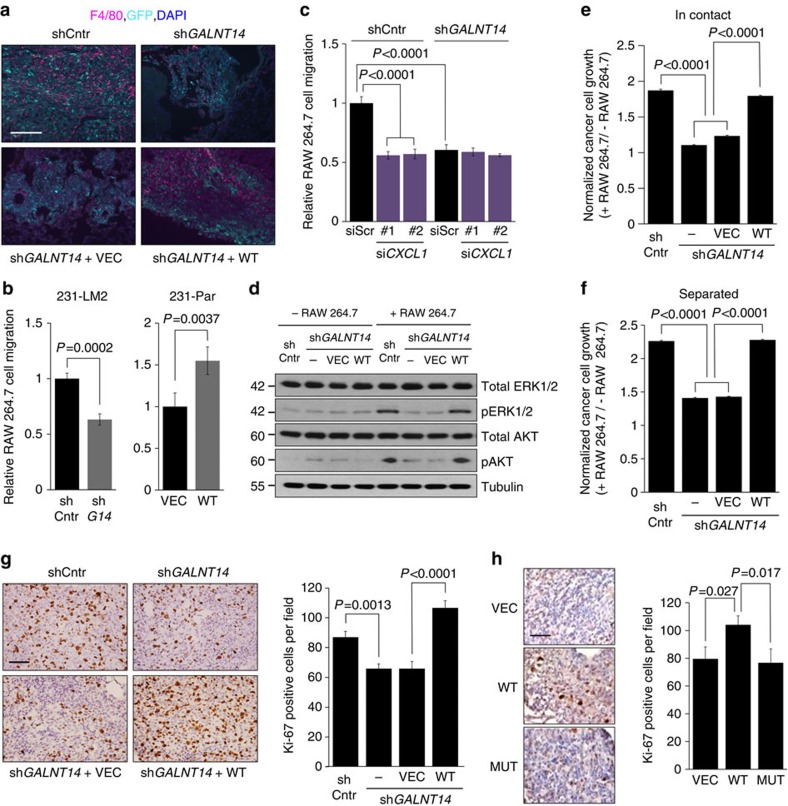
GALNT14 enables BCCs to modify and exploit the lung microenvironment for their metastatic outgrowth. (**a**) Immunofluorescent staining of frozen lung sections from mice injected with the indicated 231-LM2 cells by using an antibody against a macrophage marker F4/80. Representative images from at least three independent lung samples are shown. F4/80 (magenta), GFP (cyan), DAPI (blue). GFP-positive cells represent cancer cells. Scale bar, 100 μm. (**b**) Relative RAW 264.7 cell transwell migration by CM from the indicated 231-LM2 (left) and 231-Par cells (right). *n*=6. (**c**) Similar experiments as in **b** except that CM were collected from shCntr and sh*GALNT14*-expressing 231-LM2 cells transfected with scrambled (siScr) and two independent siRNAs against *CXCL1*. *n*=9. (**d**) The role of GALNT14 in macrophage-stimulated growth of BCCs. The indicated 231-LM2 cells were cultured alone or co-cultured with RAW 264.7 cells but separated by a permeable membrane. Cell lysates were then subjected to immunoblotting analysis by using antibodies against the indicated proteins. Western blot images are representative of three independent experiments. (**e**,**f**) The indicated 231-LM2 cells were co-cultured in contact with (**e**) or separated from RAW 264.7 cells by a permeable membrane (**f**) for 3 days. The growth of 231-LM2 cells in the presence of RAW 264.7 was measured by luminescent signals and normalized to that in the absence of RAW 264.7. *n=*6 (**e**) and 9 (**f**). (**g**,**h**) Paraffin-embedded lung sections from mice injected with the indicated 231-LM2 (**g**) and 231-Par (**h**) cells were subjected to the immunohistochemistry with an antibody against Ki-67. Left: Quantification of Ki-67 staining. *n=*9. Right: Representative Images. Scale bar, 50 μm. *P* values were calculated using two-tailed unpaired Student's *t*-test. Results represent mean±s.e.m. (error bars).

In addition to initiating metastatic colonies, lung-metastatic BCCs recruit various types of stromal cells to create a favourable microenvironment for their continuous growth in the lung^1^,^41^. Thus, we explored the role of GALNT14 in this process. Lung nodules formed by control 231-LM2 cells exhibited extensive staining for a macrophage marker (F4/80). Moreover, we observed that these nodules were associated with M2-type macrophages (CD206^+^), which are known for their tumour-promoting functions (Supplementary Fig 7a). In contrast to control nodules, lesions formed by *GALNT14*-knockdown 231-LM2 cells contained a markedly reduced number of macrophages, which was restored in those formed by rescued cells (Fig. 6a). Likewise, lung metastases formed by 231-Par cells overexpressing wild-type GALNT14 exhibited increased macrophage infiltration compared with the control lesions (Supplementary Fig. 7b). Supporting our *in vivo* data, conditioned media (CM) from *GALNT14*-silenced 231-LM2 cells and overexpressing 231-Par cells impaired and facilitated the transwell migration of RAW 264.7 mouse macrophage cells (RAW cells), respectively (Fig. 6b). Taken together, our data indicate that GALNT14 plays a role in the recruitment of macrophages to the site of metastases, most likely by modulating the secretion of cyto/chemokines.

To identify a secreted factor(s) that mediate(s) RAW cell recruitment by GALNT14-expressing BCCs, we compared the levels of 80 cyto/chemokines between CM from control and *GALNT14*-knockdown 231-LM2 cells. Intriguingly, *GALNT14* knockdown reduced the production of several cyto/chemokines, including CXCL1 (Supplementary Fig. 7c), which is known to function in myeloid cell recruitment^42^,^43^. Notably, while the GALNT14-dependent increase in CXCL1 production was confirmed by ELISA, no change in its transcript level was observed (Supplementary Fig. 7d,e). These findings indicate that GALNT14 regulates CXCL1 at the post-transcriptional level. More importantly, CM from *CXCL1*-silenced 231-LM2 cells considerably reduced RAW cell migration while *CXCL1* knockdown in sh*GALNT14*-expressing 231-LM2 cells caused no further decrease (Fig. 6c and Supplementary Fig. 7f). Conversely, the addition of recombinant CXCL1 to the CM from *GALNT14*-depleted 231-LM2 cells recovered RAW cell migration to the level similar to what we observed with control cells (Supplementary Fig. 7g). Thus, our data support that GALNT14-expressing BCCs may enhance macrophage recruitment by promoting CXCL1 production.

Once stromal cells are recruited to metastatic sites by BCCs, they produce various growth-stimulating factors that can be exploited by metastatic BCCs. Thus, we examined whether GALNT14 allows BCCs to better respond to macrophage-derived growth stimuli. Co-culture with RAW cells led to activation of the ERK1/2 and AKT pathways in 231-LM2 cells, which are associated with BCC survival and growth. However, RAW cell-mediated activation of these pathways was not observed in *GALNT14*-knockdown 231-LM2 cells (Fig. 6d). Accordantly, co-culture with RAW cells enhanced the growth of control but not *GALNT14*-knockdown 231-LM2 cells (Fig. 6e). Similar results were observed with CN34-LM1 and MCF7 cells (Supplementary Fig. 8a,b). Furthermore, macrophage-induced growth of 231-LM2 cells was maintained even under the conditions in which the RAW and 231-LM2 cells were separated by a permeable membrane^44^, indicating that this effect is mediated by secreted factor(s) from RAW cells (Fig. 6f). Supporting our *in vitro* data, immunohistochemical analysis with a proliferation marker Ki-67 showed reduced proliferation of *GALNT14*-silenced 231-LM2 cells, whereas wild-type GALNT14-overexpressing 231-Par cells exhibited increased proliferation in the lung (Fig. 6g,h).

Collectively, our data suggest that GALNT14-expressing BCCs secure successful metastatic growth not only by modifying the lung microenvironment but also by exploiting macrophage-derived signals for their growth.

### GALNT14 promotes BCC growth by activating FGF signalling

To investigate the molecular mechanisms by which GALNT14 enhances the macrophage-stimulated growth of BCCs, we further inspected our RNA-seq data (Fig. 5a). Our findings indicated that the macrophage-induced BCC growth is mediated by a secreted factor(s) and involves the activation of AKT and ERK signalling. Thus, we searched for the signalling pathway(s) that is (are) triggered by cell-surface receptors and subsequently activate(s) the AKT and or ERK pathways. Interestingly, our analyses revealed fibroblast growth factor (FGF)-responsive genes including ets variant 1 *(ETV1)* and sprouty homologue 1 (*SPRY1)* as downstream targets of GALNT14 (Fig. 5a and Supplementary Fig. 9a–c). The activation of FGF signalling is initiated by the binding of FGF ligands to their cognate FGF receptors (FGFRs) and often involves the activation of several downstream signalling cascades, such as the AKT and ERK pathways^45^,^46^. In cancer, it has been suggested that deregulated FGF signalling play both cancer-promoting and suppressive roles^47^,^48^,^49^,^50^. However, the GALNT14-mediated regulation of FGF signalling and its functional role in the lung metastasis of breast cancer remains unknown.

**Figure 7:**
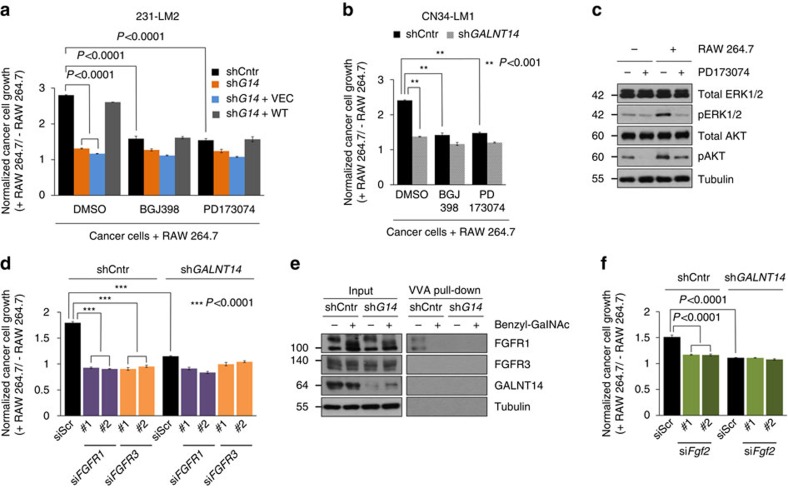
GALNT14 mediates macrophage-stimulated BCC growth in the lung parenchyma via activation of FGF signalling. (**a**,**b**) Comparison of macrophage-stimulated growth of the indicated 231-LM2 (**a**) and CN34-LM1 (**b**) cells in the absence or presence of pan-FGFR inhibitor BGJ398 (100 nM) or FGFR1/3-specific inhibitor PD173074 (100 nM). The inhibitors were treated for 12 h before co-culture and relative cell growth was analysed after 3 days. *n*=6. (**c**) The indicated 231-LM2 cells were cultured in the absence or presence of RAW 264.7 cells with or without PD173074 and the phosphorylation of ERK1/2 and AKT was analysed. (**d**) 231-LM2 cells, transfected with scrambled or two independent siRNAs against *FGFR1* or *FGFR3*, were grown in the absence or presence of RAW 264.7 and their relative growth rates were analysed. *n=*6. (**e**) O-glycosylation of endogenous FGF receptor 1. Lysates from the indicated 231-LM2 cells were subjected to VVA pull-down assays as in Fig. 4f, followed by the western blot analyses with FGFR1 and FGFR3 antibodies. 1 mM benzyl-GalNAc was added to the media 24 h before pull-down where indicated. (**f**) The indicated 231-LM2 cells were co-cultured with RAW 264.7 cells expressing scrambled siRNA (siScr) or two independent si*Fgf*2 RNAs and the relative growth of 231-LM2 cells were analysed. *n=*6. *P* values were calculated using two-tailed unpaired Student's *t*-test. Data are mean±s.e.m. Western blot images in **c**,**e** are representative of three independent experiments.

To test the possibility that GALNT14 supports the macrophage-stimulated growth of BCCs by increasing responsiveness to macrophage-derived FGFs, we examined whether inhibiting FGF signalling abolishes the macrophage-induced growth of GALNT14-expressing BCCs. Treatment with the pan-FGFR kinase inhibitor BGJ398 abrogated the macrophage-stimulated growth of control 231-LM2 and CN34-LM1 cells, while no significant effect was observed with *GALNT14*-silenced cells (Fig. 7a,b). Interestingly, the FGFR1/3-specific inhibitor PD173074 not only exhibited a similar inhibitory effect on macrophage-induced BCC growth as BGJ398 (Fig. 7a,b) but also caused a loss of pERK1/2 and pAKT (Fig. 7c). These data indicate that GALNT14-mediated activation of FGF signalling mainly engages FGFR1 and 3. Consistent with this, the knockdown of *FGFR1* and *3* (Supplementary Fig. 9d,e) completely abrogated macrophage-induced growth of control 231-LM2 cells while no further decrease was observed with *GALNT14*-silenced 231-LM2 cells (Fig. 7d).

Given these findings, we then examined whether the activation of FGF signalling by GALNT14 involves the glycosylation of FGFR1 and/or 3. We observed GALNT14-dependent O-GalNAcylation of both exogenously-expressed and endogenous FGFR1, but not FGFR3 (Fig. 7e and Supplementary Fig. 9f), which further supports the GALNT14-mediated activation of FGF signalling in BCCs.

Next, we sought to identify macrophage-derived FGF(s) that function(s) as a growth-stimulating factor for GALNT14-expressing BCCs. To this end, we tested whether FGF2 (b-FGF), known to be secreted by tumour-associated macrophages^51^, is involved in this process. Notably, knockdown of *Fgf2* in RAW cells reduced the macrophage-induced growth of control, but not *GALNT14-*knockdown 231-LM2 cells (Fig. 7f and Supplementary Fig. 9g). This result suggests that macrophage-derived FGF2 at least plays a partial role in stimulating the growth of GALNT14-expressing BCCs although we cannot exclude the contribution of other FGFs.

Collectively, our data suggest that O-GalNAcylation of FGFR1 by GALNT14 primes lung-metastatic BCCs for the activation of FGF signalling, conferring their successful growth upon receiving macrophage-derived FGFs (for example, FGF2) in the lung parenchyma.

### The KRAS-PI3K pathway upregulates *GALNT14* in BCCs

Having uncovered the mechanisms underlying GALNT14-mediated breast cancer metastasis to the lung, we next sought to identify the upstream regulators that are responsible for the elevated *GALNT14* expression in lung-metastatic BCCs.

**Figure 8:**
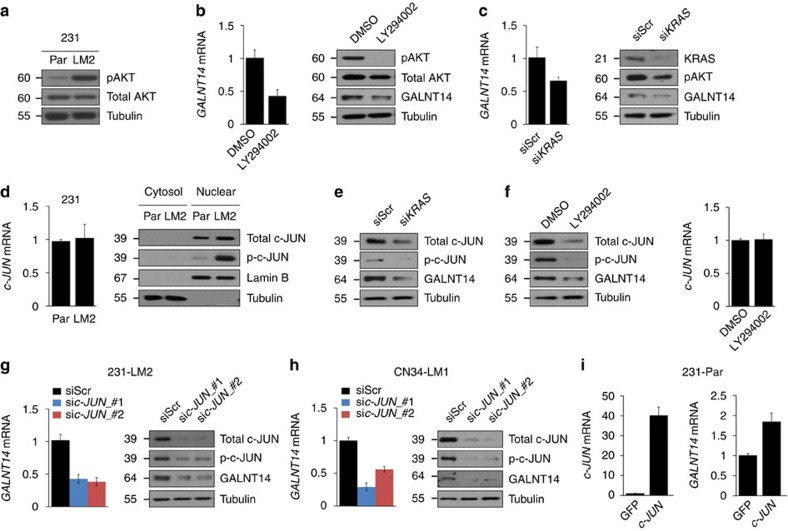
Activated KRAS-PI3K-c-JUN signalling is responsible for elevated *GALNT14* expression in lung-metastatic BCCs. (**a**) Comparison of total and phosphorylated AKT (pAKT) levels between 231-Par and LM2 cells. (**b**,**c**) Relative *GALNT14* transcript (left) and protein (right) levels in 231-LM2 cells treated with DMSO or 50 μM of PI3K inhibitor LY294002 for 16 h (**b**) or transfected with scrambled (Scr) or siRNA pool against *KRAS* (**c**). Cell lysates were then subjected to qRT-PCR or western blot analysis. (**d**) Relative levels of *c-JUN* mRNA (left) and total and phosphorylated c-JUN at S63/73 in cytosolic and nuclear fraction (right) of 231-Par and LM2 cells. (**e**,**f**) Comparison of total and phosphorylated c-JUN as well as GALNT14 protein levels in 231-LM2 cells transfected with scrambled or *KRAS* siRNAs (**e**) or treated with LY294002 (**f**). Relative *c-JUN* mRNA levels are shown on the right. (**g**,**h**) Changes in *GALNT14* mRNA (left) and protein levels (right) upon knockdown of *c-JUN* in 231-LM2 (**g**) or CN34-LM1 (**h**). Cells were transfected with scrambled siRNA (siScr) or two independent siRNAs against *c-JUN* (sic-JUN_#1 and_#2). c-*JUN* knockdown was confirmed by western blotting. (**i**) 231-Par cells were transfected with GFP or *c-JUN* expression construct and relative *c-JUN* (left) and *GALNT14* (right) mRNA levels were analysed by qRT-PCR. Results represent mean±s.e.m. (error bars). Western blot images in **a**–**h** are representative of two to three independent experiments.

Metastatic cancer cells often exhibit the activation of several signal transduction pathways, which is generally associated with the altered expression of downstream target genes. To identify the upstream pathway(s) that contributes to transcriptional induction of *GALNT14* in lung-metastatic BCCs, we searched for signalling pathways that are hyper-activated in 231-LM2 compared with 231-Par cells. We found a dramatic increase in the phospho-AKT level in 231-LM2, which suggested activated PI3K signalling in these cells (Fig. 8a). In addition, the PI3K inhibitor LY294002 reduced the *GALNT14* transcript levels (Fig. 8b) while the inhibition of NOTCH or TGF signalling pathways, previously shown to be activated in lung-metastatic BCCs^29^,^52^, did not alter its expression (Supplementary Fig. 10a,b). These data suggest a specific role of the PI3K pathway in the upregulation of *GALNT14* in lung-metastatic BCCs.

The PI3K pathway can be triggered by various mechanisms, including the activation of KRAS^53^,^54^. Thus, we examined whether KRAS controls *GALNT14* expression by activating the PI3K pathway. *KRAS* knockdown led to reduced phospho-AKT as well as *GALNT14* transcript levels (Fig. 8c), supporting the role for the activated KRAS-PI3K pathway in *GALNT14* expression in lung-metastatic BCCs.

The KRAS-PI3K pathway modulates the activities of several transcription factors, leading to changes in the expression of downstream target genes^54^,^55^. Therefore, we searched for a potential transcription factor that is activated by the KRAS-PI3K signalling cascade and regulates *GALNT14* expression. The examination of the *GALNT14* promoter region uncovered two c-JUN-binding sites located approximately 2.4 kb upstream and 650 bp downstream of transcription start site. This caught our attention because the KRAS-PI3K pathway has been shown to regulate both the transcriptional activity and stability of c-JUN by modulating its phosphorylation^54^,^56^. Notably, the activation of the PI3K pathway in 231-LM2 coincided with a marked increase in phosphorylated c-JUN at Ser63/Ser73, the active form of the protein (Fig. 8d). This finding led us to test whether the KRAS-PI3K pathway governs *GALNT14* expression by regulating the c-JUN protein. *KRAS* knockdown and the inhibition of PI3K activity decreased total and phospho-c-JUN level in both 231-LM2 and CN34-LM1 cells (Fig. 8e,f and Supplementary Fig. 10c,d). Moreover, *c-JUN* knockdown reduced *GALNT14* transcription in these cells (Fig. 8g,h) while the overexpression of c-JUN in 231-Par cells increased its expression (Fig. 8i). This suggests that c-JUN positively regulates *GALNT14* expression at the transcription level.

Collectively, our findings demonstrate that the hyper-activated KRAS-PI3K pathway in lung-metastatic BCCs promotes the c-JUN-mediated transcription of *GALNT14*.

## Discussion

**Figure 9:**
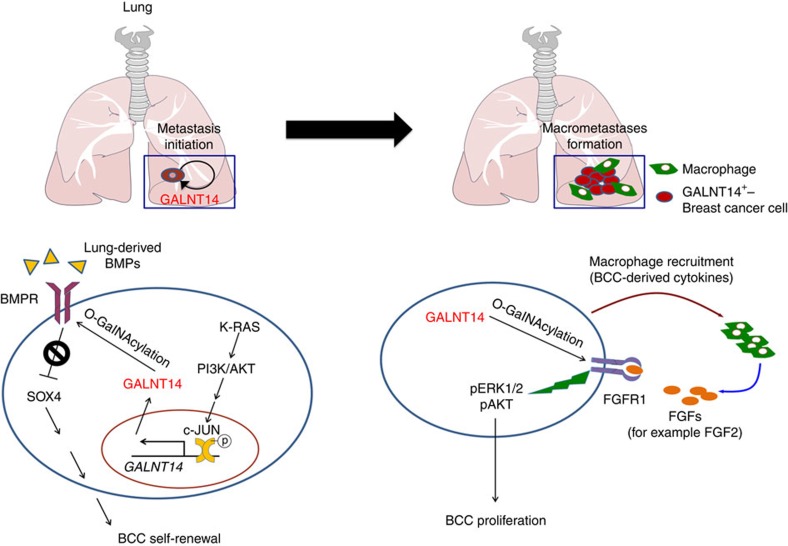
Proposed working model of GALNT14-mediated lung-specific metastasis of breast cancer. Left: the KRAS-PI3K pathway, which is activated in BCCs with high lung-metastatic potential, induces the c-JUN-mediated transcriptional upregulation of *GALNT14*. GALNT14-expressing BCCs possess the ability to self-renew against lung-derived BMPs, potentially via GALNT14-mediated BMPR O-GalNAcylation, which results in the attenuation of BMP responsiveness and therefore an increase in *SOX4* expression. As a result, GALNT14-expressing BCCs can initiate metastatic colonies in the lung parenchyma. Right: To establish macrometastases, GALNT14-expressing BCCs secrete chemokines, which creates a favourable microenvironment by stimulating macrophage infiltration. Furthermore, the O-GalNAcylation of FGFR1 by GALNT14 enables BCCs to exploit macrophage-derived FGFs (for example, FGF2), ensuring their continuous proliferation within the lung microenvironment.

Accumulating evidence suggests that while there exists a class of genes that enhances the general metastatic potential of cancer cells, others contribute to organ-specific metastasis^1^,^8^,^29^,^57^. The latter class of genes generally plays a role in the late stages of the metastatic process, such as the initiation of metastatic colonies and their subsequent outgrowth into macrometastases. In the present study, we revealed that GALNT14 specifically promotes breast cancer metastasis to the lung by accelerating both of these events in the lung parenchyma (Fig. 9).

The initiation of metastasis at a distal site is an extremely challenging task for cancer cells due to a number of anti-metastatic signals produced by the destination organ. Thus, it is thought that metastasis-initiating cells possess stem cell-like properties, including the ability to self-renew against these inhibitory signals. Consistent with this notion, our study demonstrated that GALNT14 allows BCCs to self-renew in the presence of the lung-derived anti-metastatic signals BMPs, by suppressing BMP signalling through BMPR glycosylation. Recently, a study by Gao *et al*. indicated that lung-metastatic BCCs secrete an increased level of a BMP-antagonist called COCO, which blocks the binding of lung-derived BMPs to their cognate receptors and thus, facilitates the reactivation of BCCs from dormancy^30^. Therefore, it is plausible that BCCs overcome the inhibitory effect of BMPs on self-renewal by a combination of these two mechanisms. Further evidence supporting the stem cell-like properties of GALNT14-expressing BCCs include: (1) GALNT14 induces *SOX4* expression, which subsequently increases transcription of stem cell-associated factors *SOX2* and *OCT4*; and (2) GALNT14 augments mammary tumour initiation when a limited number of BCCs are implanted.

The establishment of metastatic colonies requires constant communication between cancer cells and stromal components in the secondary organ. Our studies indicated that GALNT14 promotes macrophage recruitment by enhancing the production of CXCL1. Based on our data demonstrating that GALNT14 does not upregulate CXCL1 at the transcriptional level, we speculate that the enhanced CXCL1 production could be due to increased stability of CXCL1 by GALNT14-mediated O-glycosylation. Supporting this idea, the O-glycosylation of several cytokines has been shown to increase their stability^58^. However, the exact molecular mechanism through which GALNT14 regulates the production of CXCL1 warrants further investigation.

In addition to its role in macrophage recruitment by BCCs, our study demonstrated that GALNT14 enables BCCs to exploit macrophage-derived FGFs for their continuous growth in the lung. The supporting evidence includes: (1) the association between increased macrophage infiltration and proliferation of GALNT14-expressing BCCs *in vivo*; (2) the requirement of GALNT14 in macrophage-induced growth of BCCs; (3) the ablation of this growth advantage upon the pharmacological inhibition or genetic knockdown of *FGFR1* and *3*; (4) the loss of macrophage-induced BCC growth upon the depletion of *FGF2* in macrophages; and (5) the GALNT14-mediated glycosylation of FGFR1. Of note, while *FGFR3* knockdown showed similar results as that of *FGFR1*, we only observed the O-GalNAcylation of FGFR1 but not FGFR3. Aside from technical limitations, such as the sensitivities of antibodies, we speculate that macrophage-mediated cancer cell growth may involve the FGFR1-FGFR3 heterodimer, and this effect could be modulated by the O-GalNAcylation of FGFR1. Supporting this idea, the heterodimerization of different FGFRs^59^ and the role of O-GalNAcylation in the dimerization of other cell-surface receptors have been reported^36^,^60^.

The biological functions of GALNTs are linked to their enzymatic activities. Accordingly, our data suggest that GALNT14 may function through the glycosylation of BMPR1A (ALK3) and FGFR1, which results in the suppression and activation of the downstream signalling pathway, respectively. Consistent with this possibility, a previous study reported that ALK3 and ActR-IIB are O-GalNAcylated by *Xenopus* Galntl-1 and that ActR-IIB glycosylation prevents its dimerization with BMPRI^36^. In addition, the O-GalNAcylation of FGFR2 by an unknown GALNT has been shown to trigger the activation of downstream signalling^35^. The biochemical mechanisms by which the GALNT14-stimulated O-GalNAcylation of FGFR1 activates the downstream cascade are currently under investigation. On the basis of previous studies showing that the N-glycosylation of FGFR1 and FGFR2 contributes to enhanced ligand binding and proper localization of the receptors, respectively^61^,^62^, we presume that O-GalNAcylation may activate FGF signalling by similar mechanism. Alternatively, the O-GalNAcylation of FGFR1 may facilitate the formation of receptor dimers as mentioned above.

The differential expression patterns of GALNT isozymes have been reported. Although a few studies have shown that the differential expression of microRNAs can regulate the expression of certain *GALNT*s^63^,^64^,^65^, the molecular mechanisms controlling the expression of different GALNT members are poorly understood. The current study revealed that the activated KRAS-PI3K-c-JUN pathway is responsible for the upregulation of *GALNT14*, providing a mechanistic insight into its elevated expression in lung-metastatic BCCs.

Supporting our experimental data, clinical data analysis revealed that the elevated level of *GALNT14* in primary breast tumours specifically predicts lung relapse, suggesting the potential use of *GALNT14* expression in identifying patients with high risks of pulmonary metastases. Recently, GALNT14 has been shown to be associated with neuroblastoma, hepatocarcinoma, and lung adenocarcinoma^21^,^66^,^67^. Furthermore, elevated *GALNT14* expression has been proposed as a biomarker for TRAIL-sensitive tumours^60^. However, we did not observe changes in TRAIL sensitivity upon the ectopic expression of *GALNT14* in 231-Par cells (unpublished data), which is consistent with other studies indicating that the GALNT14-mediated increase in TRAIL sensitivity is a tumour type-dependent phenomenon.

In conclusion, our study not only uncovered a novel function of GALNT14 in the lung metastasis of breast cancer and delineated the underlying molecular mechanisms but also suggests that this enzyme can be a potential therapeutic target for breast cancer treatment. Further studies on the function of other GALNT members in different subtypes of breast cancers (Supplementary Data set 1) as well as other cancer types will provide further insight into the diverse roles of GALNTs in cancer progression and metastasis.

## Methods

### Materials

TGFβR inhibitor (SB431542), NOTCH inhibitor (DAPT), BMPR inhibitor (DMH1), methylcellulose and O-glycosylation inhibitor (Benzyl 2-acetamido-2-deoxy-α-D-galactopyranoside) were purchased from Sigma-Aldrich (St Louis, MO). For PI3K inhibition, LY294002 was obtained from Calbiochem (San Diego, CA). For FGFR inhibition, BGJ398 and PD173074 were purchased from Selleckchem (Houston, TX). Recombinant human BMP4 and CXCL1 were purchased from Peprotech (Rocky Hill, NJ).

### Cell culture

MCF7, CN34-LM1, MDA-MB-231 and their metastatic derivatives were kindly provided by Dr Joan Massague (Memorial Sloan Kettering Cancer Center), and Dr Sohail Tavazoie (Rockefeller University)^6^. Human umbilical vein endothelial cells (HUVEC) were purchased from LONZA (Basel, Switzerland) and cultured in EGM-2 bullet kit media (LONZA)^5^. 4T1 derivatives and RAW 264.7 cells were provided by Drs Fred Miller (University of Michigan) and Suk-Jo Kang (KAIST), respectively. CN34-LM1 cells were cultured in M199 medium containing 2.5% FBS, 10 μg ml^−1^ insulin, 0.5 μg ml^−1^ hydrocortisone, 20 ng ml^−1^ EGF and 100 ng ml^−1^ cholera toxin. All the other cells were cultured in in DMEM supplemented with 10% FBS. The cell lines were recently authenticated by DNA fingerprinting analysis and regularly tested for mycoplasma contamination.

### Animal studies

All experiments using animals were done in accordance with procedures approved by the Korean Advanced Institute of Science and Technology Institutional Animal Care and Use Committee (IACUC). Age-matched (5 to 6 weeks) female NOD/SCID (Korea Research Institute Bioscience and Biotechnology, Republic of Korea) or BALB/c nude mice (OrientBio, Republic of Korea) were used for intravenous, intracardiac and MFP injections^8^,^52^. Tumour volume (*V*) was measured semi-weekly using calipers and calculated using the formula *V*=(length) × (width)^2^ × 0.5. For intracardiac injection, MDA231-Par (2 × 10^5^) or MDA231-BrM2 (1 × 10^5^) cells were re-suspended in 0.1 ml cold PBS and injected into the left ventricle. A bioluminescent imaging (BLI) analysis was used to measure bone or brain metastases using an IVIS Spectrum system (PerkinElmer, Waltham, MA). For experimental lung metastasis assays, MDA231-LM2 (7 × 10^4^), CN34-LM1 (6 × 10^5^) or MCF7 (2 × 10^6^) cells were intravenously injected and lung colonization was quantified using BLI. Statistical methods to predetermine sample size were not used and the experiments were not randomized. During experiments and outcome analysis, the animal group allocations were not blinded.

### Clinical data analysis

The overall DMFS was conducted through KM plotter for breast cancer in http://kmplot.com/analysis website using 2014 version of database^22^. Among 20 *GALNT*s, only 18 *GALNT* probes were suitable for DMFS analysis. The probes used in analyses are: *GALNT1* (201724_s_at), *GALNT2* (217788_s_at), *GALNT3* (203397_s_at), *GALNT5* (229555_at) *GALNT6* (228303_at), *GALNT7* (218313_s_at), *GALNT8* (220929_at), *GALNT9* (229451_at), *GALNT10* (212256_at), *GALNT11* (219013_at), *GALNT12* (218885_s_at) *GALNT13* (236536_at), *GALNT14* (219271_at), *GALNT15* (228501_at), *GALNT16* (230418_s_at), *GALNT18* (1554079_at), *GALNT19* (227434_at) and *GALNT20* (233150_at). Median expression of each *GALNT* was used to separate *GALNT* high/low patient group. MFS analysis was performed at Proboco Informatics (Ithaca, NY). EMC192, EMC286, and MSK82 (GSE12276, GSE2034 and GSE2603, respectively)^5^ were log-transformed and centred to the mean of common genes present in both GPL96 (GSE2034 and GSE2603) and GPL570 (GSE12276) microarray platforms. Finally, *GALNT14* +/− group were identified based on mean *GALNT14* expression.

### Generation of GALNT14 catalytic mutant

GALNT14 mutant was generated by substituting three amino acid residues in glycosyltransferase 1 (GT1) motif, which were previously identified to be critical in enzymatic activity of mouse GALNT1 and conserved among all 20 human GALNT family members^26^. The substitutions include Asp199→Asn, His201→Asp and Glu202→Gln.

### Gene knockdown and overexpression

shRNA targeting for *GALNT14* were purchased from Sigma-Aldrich. Lentiviruses were produced in 293T cells and used to infect 231-LM2 and CN34-LM1 cells. Target-specific siRNAs were obtained from Bionner (Daejeon, Korea). A scrambled siRNA (siScr) were synthesized and validated by Bionner. siGENOME SMARTpool siRNA targeting for *KRAS* were purchased from Dharmacon (Life technologies, Carlsbad, CA). siRNAs were transfected into cells by using Lipofectamine (Life technologies). Hairpin and siRNA sequences used in this study are summarized in Supplementary Table 3. The list of overexpression constructs is summarized in Supplementary Table 4.

### Co-culture assay

Co-culture assay with BCCs and RAW 264.7 cell line was performed as the following. For in contact co-culture assay, cancer cells were seeded to 24-well plate. After 24 h, the media were replaced with fresh DMEM media containing 0.2% FBS and starved for 12 h. Next, RAW 264.7 cells were suspended with DMEM media containing 0.2% FBS and added to cancer cells. For the separated co-culture experiments, we used transwell inserts (Corning, Corning, NY) with a 0.4 μm pore size. RAW 264.7 cells were seeded into transwell inserts and cultured for 3 days. Finally, cancer cell growth was analysed using Luciferase assay system (Promega, Madison, WI).

### qRT-PCR analysis

Total RNA was isolated using Qiazol (Qiagen, Valencia, CA) followed by chloroform extraction. First-strand cDNA was synthesized from 400 ng of total RNA using SuperscriptII reverse transcriptase (Life technologies) according to the manufacturer's instructions. qRT-PCR assay was performed using SYBR Green Real-time PCR Master Mix (Toyobo, Osaka, Japan) in a Real-Time PCR machine CFX96 (Biorad, Hercules, CA) using the gene-specific primer sets. Analysis of *GALNT8*, *GALNT15*, *GALNT17*, *GALNT20*, *ANGPTL4*, *B2M, MmGALNT14* and *MmB2M* mRNAs were detected using TaqMan Gene Expression Assay (Life technologies). The gene-specific primers and TaqMan assay ID used in this study were listed in Supplementary Table 5.

### Western blot analysis

Whole-cell lysates were prepared using RIPA-B lysis buffer containing 150 mM NaCl, 1 mM EDTA (pH 8.0), 20 mM Tris-Cl (pH 7.4), 1% NP-40, 0.5% sodium deoxycholate, 1% Triton X-100, 1 mM Na_3_VO_4_, 5 mM NaF, 10% glycerol and protease inhibitor cocktail (Roche, basel, Switzerland). For preparation of nuclear extracts, BCCs were washed using cold PBS and lysed with nuclear extraction Buffer A containing 20 mM HEPES-KOH pH 7.9, 10 mM KCl, 1 mM EDTA, 5 mM NaF, 10 mM β-glycerophosphate, 1 mM Na_3_VO_4_, 1 mM DTT, 1% NP-40 and protease inhibitor cocktail, followed by incubation on ice for 15 min. The nuclear pellets were lysed in hypotonic Buffer B containing 20 mM HEPES-KOH pH 7.9, 0.4 M NaCl, 1 mM EDTA, 5 mM NaF, 10 mM β-glycerophosphate, 1 mM Na_3_VO_4_, 1 mM DTT and protease inhibitor cocktail. Equivalent amounts of proteins were measured using BCA protein assay kit (Sigma-Aldrich) and separated by electrophoresis on SDS-PAGE and then transferred to a polyvinylidene difluoride (PVDF) membrane (Roche). Primary antibodies used in this study are summarized in Supplementary Table 6. The original scan of western blots are provided as Supplementary Fig. 11.

### Genome-wide RNA-seq analysis

RNAs isolated from MDA231-LM2 cells expressing shCntr or sh*GALNT14* and MDA231-Par cells expressing pBabe-Hygro vector or GALNT14 were subjected to enrichment of polyA+ RNA using Dynabeads Oligo (dT)25 (Invitrogen)^68^. Strand-specific RNA-seq libraries were prepared from the polyA+ RNA^68^. The RNA-seq libraries were sequenced using single-end methodology with a length of 50 nt (SE50) (two replicates each) using an Illumina HiSeq 2500. The computational pipeline to analyse strand-specific RNA-Seq data includes the following steps: RNA-Seq reads were aligned to the human reference genome (NCBI 37, hg19) using the spliced read aligner TopHat version 1.4.0 (ref. 69). The bam files from TopHat were assembled using Cufflinks^70^. The individual transcript assemblies from multiple RNA-Seq libraries are assembled into one master transcriptome using Cuffmerge. This step is required for differential expression analysis of the assembled transcripts. Finally, using Cuffdiff comparisons of expression levels of genes from the different conditions was performed. Heat map was generated by using online program ‘Matrix2png'; http://www.chibi.ubc.ca/matrix2png/bin/matrix2png.cgi.

### Oncosphere assays

Oncosphere assays were performed as previously described^30^. Briefly, cancer cells were collected by using scraper and filtered using 40 μm pore cell strainer (BD Biosciences). 0.5–2.5 × 10^4^ single cells were seeded into 6-well ultra-low binding plates (Corning) and cultured in Human mammary epithelial cell (HuMEC) media (Life Technologies) containing 20 ng ml^−1^ EGF, 5 μg ml^−1^ insulin, 10 μg ml^−1^ heparin, 40 ng ml^−1^ basic FGF, 1X B27 supplement without vitamin A and 0.02% methylcellulose for 7 days. Spheres over 100 μm diameter were counted and pictured using Nikon ECLIPSE TS100 microscope.

### Vicia villosa lectin (VVA) ELISA

For VVA ELISA, whole-cell lysates were prepared using RIPA-B lysis buffer. RIPA-B was exchanged with PBS using centrifugal filter units (Merck Millipore) which was repeated 4 times. 40 μg total proteins were measured using BCA protein assay kit and coated onto the ELISA microplate (Greiner bio-one, Neuburg, Germany) for 16 h at 4 °C. The total bound proteins were washed with PBS containing 0.05% Tween 20 and treated with blocking solution (Vector Labs, Burlingame, CA) for 2 h at 4 °C. Each well was washed three times and added with 10 μg ml^−1^ biotinylated VVA (Vector Labs) for 16 h at 4 °C. Target proteins bound to biotinylated VVA were stained using Vectastain ABC kit (Vector Labs). Absorbance was measured using a microplate spectrophotometer (Berthold technologies, Wildbad, Germany) at 405 nm.

### VVA pull-down assay

Cell lysates were collected using cell lysis buffer containing 120 mM NaCl, 40 mM Tris-Cl (pH 7.4), 1 mM EDTA (pH 8.0), 0.3% Chaps, 1 mM Na_3_VO_4_ and protease inhibitor cocktail. 1 mg total proteins were subjected to pull-down assay with agarose bound VVA (Vector Labs). After incubation in rotator for 16 h at 4 °C, protein-agarose bound VVA mixture was washed four times using washing buffer containing 120 mM NaCl, 40 mM Tris-Cl (pH 7.4), 1 mM EDTA, 1 mM Na_3_VO_4_ and protease inhibitor cocktail. After centrifugation, the pellet was added to 5X SDS buffer containing 60 mM Tris-Cl (pH 6.8), 25% glycerol, 2% SDS, 0.1% bromophenol blue and 5% β-mercaptoethanol and denatured by boiling. Finally target proteins were detected by western blotting.

### Immunohistochemistry and immunofluorescent staining

Metastatic lesions were confirmed by histological analysis. At the end of animal experiment, mice were killed and perfused with PBS through the left ventricle before tissues were extracted. Extracted tissues were fixed with 4% paraformaldehyde, paraffin-embedded or snap frozen in liquid nitrogen, and stained with hematoxylin and eosin. For the Ki-67 staining, paraffin-embedded sections were stained with Ki-67 antigen antibody (Vector Labs) and detected with Vectastain ABC kit (Vector Labs). For immunofluorescent staining, 10-μm-thick cryostat sections were stained with antibody against mouse F4/80 at 1:500 dilution (MCA_497GA, Abd Serotec, Raleigh, NC) followed by incubation with rhodamine-conjugated secondary antibody (Jackson ImmunoResearch, West Grove, PA). For CD206 staining, mouse CD206-Alexa fluor 594 (No. 141726, Biolegend, San Diego, CA) was used at 1:100 dilution. Images were taken from fluorescent microscope (Zeiss).

### Cell viability and anoikis assays

For cell viability assay, 500-1,000 cells were seeded into 96-well plates and incubated for 3 or 6 days. Relative cell growth was measured using CellTiter-Glo Luminescent Cell Viability Assay (Promega) in 96-well plates according to the manufacturer's instructions. The luminescence was determined by a microplate luminometer (Berthold technologies). For anoikis assay, 5,000 cells were plated onto 96-well ultra-low binding plates (Corning) and were incubated in serum-free HuMEC media (Life Technologies) containing 0.2% methylcellulose. Thereafter, total cells were collected and mixed the cell suspension at 1:1 with 0.2% trypan blue. Dead cells were stained by trypan blue and counted using a hemocytometer.

### HUVEC and matrigel adhesion assays

For HUVEC adhesion assay, HUVEC cells were plated into 12-well gelatin coating plates and cultured in EGM-2 bullet kit media (LONZA) for 2 days. When HUVEC cells reached a >99% confluent monolayer, cancer cells were added into same plates. For matrigel adhesion assay, 12-well ultra-low binding plates (Corning) were coated with 4% growth factor reduced matrigel (BD Biosciences) at 37 °C for 3 h, followed by plating cancer cells onto matrigel-coated plates. After 30 min incubation at 37 °C, non-adherent cancer cells were washed by PBS washing. Relative numbers of adherent cancer cell were determined by using Luciferase assay system (Promega) according to the manufacturer's instructions.

### Invasion assays

50,000 cancer cells were labelled with Cell Tracker Green CMFDA dye (Invitrogen) for 30 min and starved with DMEM media containing 0.2% FBS for 16 h. These cells were then seeded onto matrigel-coated inserts and incubated for 16 h. Inserts were washed with PBS and fixed with 4% paraformaldehyde. Cells that had invaded the lower surface of the membrane were counted using fluorescent microscope.

### Wound-healing assay

231-LM2 cells were seeded in six-well plates and cultured until 95% confluent. The adherent monolayer cells were scratched using 200 μl pipette tip and the plate was washed 3 times with fresh DMEM media containing 10% FBS. The scratch wounds are marked with dots using labelling pen. The plates were incubated with 5% CO_2_ at 37 °C for 16 h. Images of the scratch wounds were captured using a phase-contrast microscope. Percentage of wound-healing was calculated as the following formula: (original scratch width—scratch width after healing) (original scratch width)^−1^ × 100%.

### RAW 264.7 cell migration assays

231-LM2 cells were transfected with siRNAs against *CXCL1* and seeded in six-well plates. When cancer cells reached a >95% confluent monolayer, the culture media were exchanged using DMEM media containing 0.2% FBS. After 48 h, CM were placed into the lower chamber of plate. 3 × 10^5^ RAW 264.7 cells were labelled with Cell Tracker Green CMFDA dye and place onto the FluoroBlok (Corning) transwell insert. After 9 h incubation at 37 °C, migrated RAW 264.7 cells were fixed with 4% paraformaldehyde and counted. For human cytokine treatment experiment, recombinant human CXCL1 was added to CM before migration. Migrated cells were captured and counted using fluorescent microscope.

### Cytokine antibody array

To generate CM, 1 × 10^6^ MDA231-LM2 cells were seeded on six-well plates. The next day culture media were replaced with DMEM containing 0.2% FBS. After 48 h, CM was collected and used in cytokine antibody array. The experiment was performed according to manufacturer's instruction. The complete array map (Array C5) can be found in http://www.raybiotech.com/c-series-human-cytokine-array-5-2.html.

### ELISA

Quantikine ELISA immunoassay (R&D systems) was used for quantification of secreted CXCL1 in CM of 231-Par and LM2 cell line. The experiment was performed according to manufacturer's instruction.

### Co-immunoprecipitation (Co-IP)

Cells were treated with BMP4 or TGFβ1 and lysed in 1 ml of nuclear extraction Buffer A for 15 min at 4 °C. After centrifugation, the nuclear pellets were lysed in hypotonic Buffer B for overnight at 4 °C. Equivalent amounts of nuclear proteins were diluted in immunoprecipitation buffer containing 40 mM Tris-Cl pH 7.4, 120 mM NaCl, 1 mM EDTA, 5 mM NaF, 10 mM β-glycerophosphate, 1 mM Na_3_VO_4_ and protease inhibitor cocktail and immunoprecipitated with 1 μg anti-SMAD4 (Santa Cruz Biotechnology, CA) for 16 h at 4 °C. After rocking, Protein G agarose beads (Sigma-Aldrich) were added and incubated for 4 h at 4 °C. Immunoprecipitates were pelleted by centrifugation at 200 *g* and washed three times with immunoprecipitation buffer. The bound proteins were analysed by western blotting. Information on the antibodies used in this assay are summarized in Supplementary Table 6.

### Statistical analysis

Statistical significance was determined by one-tailed Mann–Whitney test (animal experiments), log-rank test (MFS curves), or two-tailed unpaired Student's *t*-test (cell-based experiments) as indicated in the Figure legends. *P* values of <0.05 were considered as statistically significant. Data represent the mean±s.e.m. (error bars) unless indicated otherwise.

### Data availability

RNA-seq data have been deposited in the gene expression omnibus (GEO) database under access number GSE 72111. All relevant data are available from the authors on request.

## Additional information

**How to cite this article:** Song, K.-H. *et al*. GALNT14 promotes lung-specific breast cancer metastasis by modulating self-renewal and interaction with the lung microenvironment. *Nat. Commun.* **7,** 13796 doi: 10.1038/ncomms13796 (2016).

**Publisher's note:** Springer Nature remains neutral with regard to jurisdictional claims in published maps and institutional affiliations.

## Supplementary Material

Supplementary Information Supplementary Figures, Supplementary Tables, and Supplementary References

Supplementary Data 1 Analysis of distant metastasis-free survival (DMFS) and relapse-free survival (RFS). This data describes correlation between expression of GALNT members and DMFS and RFS.
