# Supplementary material for: GALNT14 promotes lung-specific breast cancer metastasis by modulating self-renewal and interaction with the lung microenvironment
Source: Nat Commun. 2016 Dec 16;7:13796. doi: 10.1038/ncomms13796 (PMC5171903; doi:10.1038/ncomms13796)
Supplement: Supplementary Information — Supplementary Figures, Supplementary Tables, and Supplementary References [file ncomms13796-s1.pdf]

a

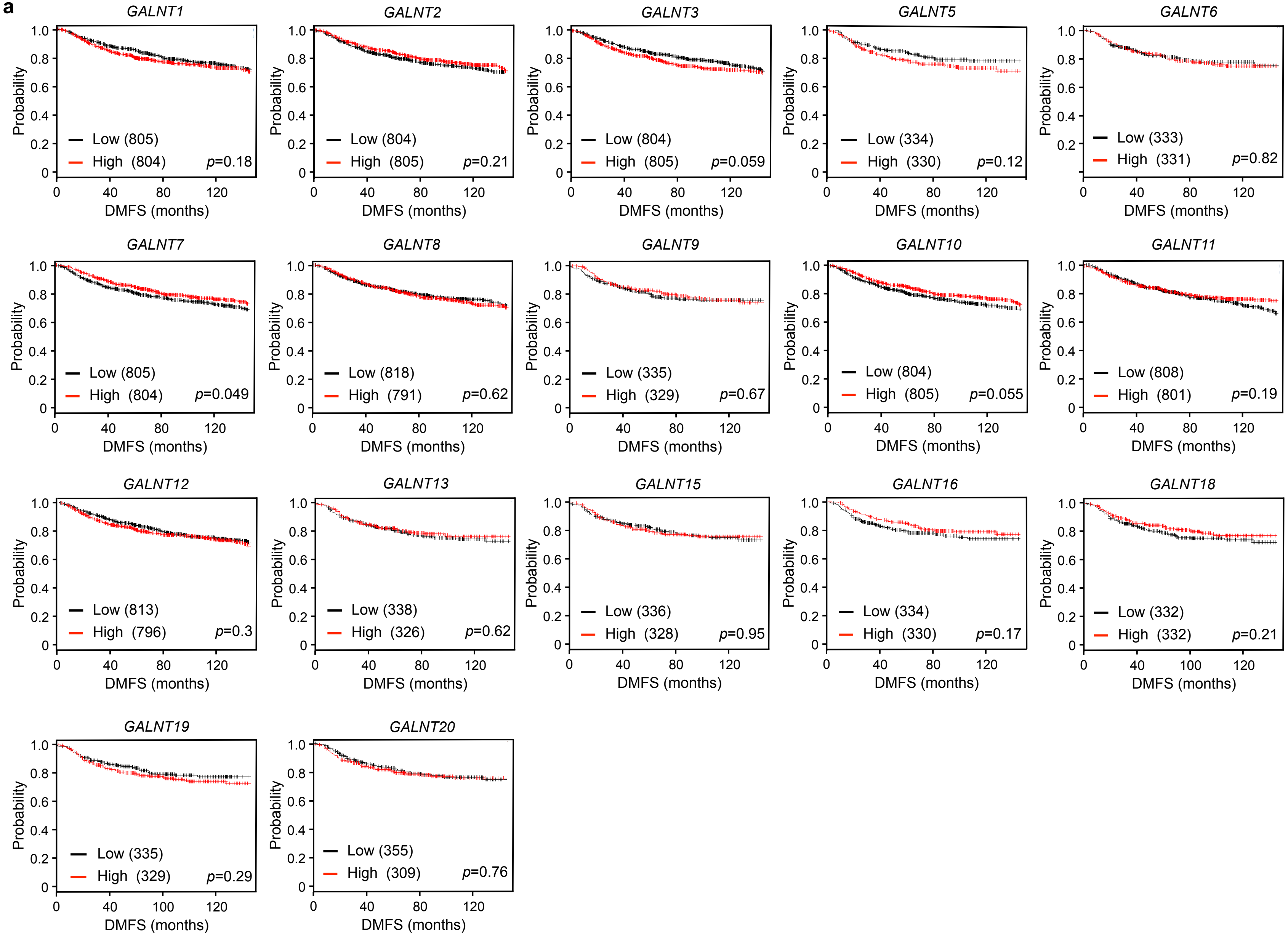
GALNT1
Low (805)
High (804)
p=0.18
GALNT2
Low (804)
High (805)
p=0.21
GALNT3
Low (804)
High (805)
p=0.059
GALNT5
Low (334)
High (330)
p=0.12
GALNT6
Low (333)
High (331)
p=0.82
GALNT7
Low (805)
High (804)
p=0.049
GALNT8
Low (818)
High (791)
p=0.62
GALNT9
Low (335)
High (329)
p=0.67
GALNT10
Low (804)
High (805)
p=0.055
GALNT11
Low (808)
High (801)
p=0.19
GALNT12
Low (813)
High (796)
p=0.3
GALNT13
Low (338)
High (326)
p=0.62
GALNT15
Low (336)
High (328)
p=0.95
GALNT16
Low (334)
High (330)
p=0.17
GALNT18
Low (332)
High (332)
p=0.21
GALNT19
Low (335)
High (329)
p=0.29
GALNT20
Low (355)
High (309)
p=0.76
Probability
DMFS (months)

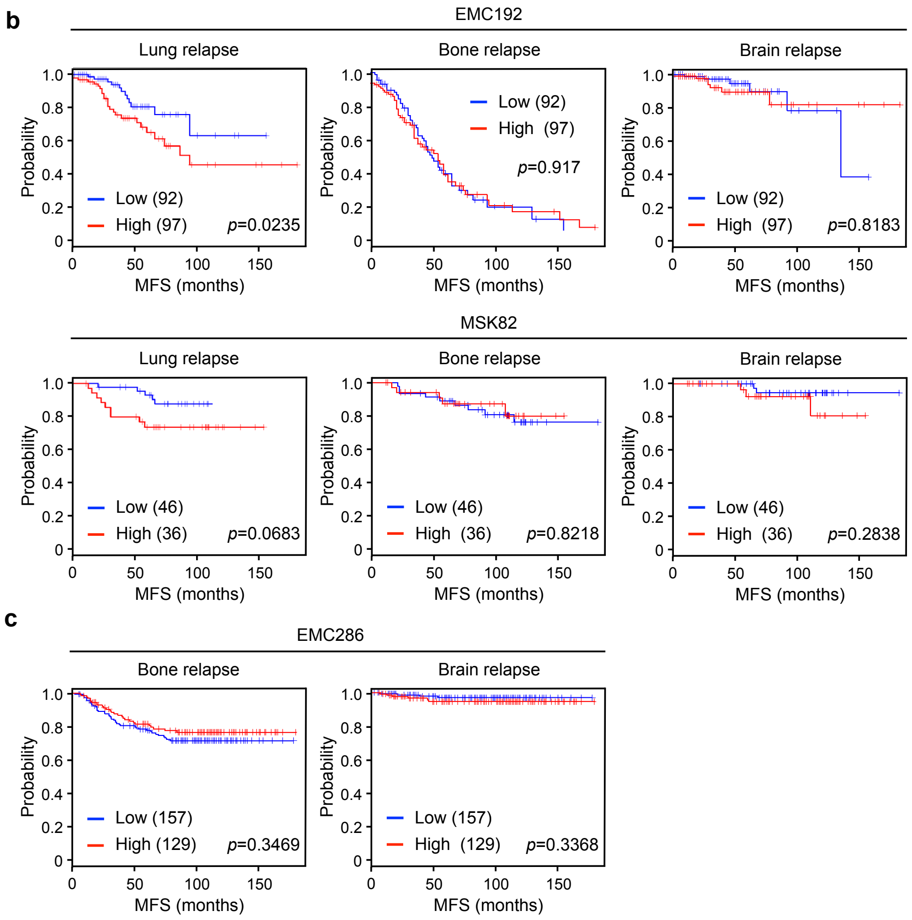
b
EMC192
Lung relapse
Bone relapse
Brain relapse
Low (92)
High (97)
p=0.0235
p=0.917
p=0.8183
MSK82
Low (46)
High (36)
p=0.0683
p=0.8218
p=0.2838
c
EMC286
Low (157)
High (129)
p=0.3469
p=0.3368
Probability
MFS (months)

Supplementary Figure 1 Expression of *GALNT14*, but not other *GALNT* members, is associated with poor distant metastasis-free survival in breast cancer patients.

(**a**) Kaplan-Meier curve representation of distant metastasis-free survival (DMFS) in breast cancer patients by using "KM plotter". Detailed methods are described in Methods section. Patients were categorized into two groups according to the median expression of the indicated *GALNT* members in their primary tumours. 18 *GALNT* family members whose expression data was available were analyzed.

(**b**) Similar analysis as in Fig. 1c except that EMC192 (top) and MSK82 (bottom) were analyzed separately. p=0.0427 by the Wilcoxon test. p=0.0683 by the log-rank test.

(**c**) Bone or brain metastasis-free survival (MFS) rates were analyzed in EMC286 cohort.

P-values were calculated using a log rank test unless indicated otherwise.

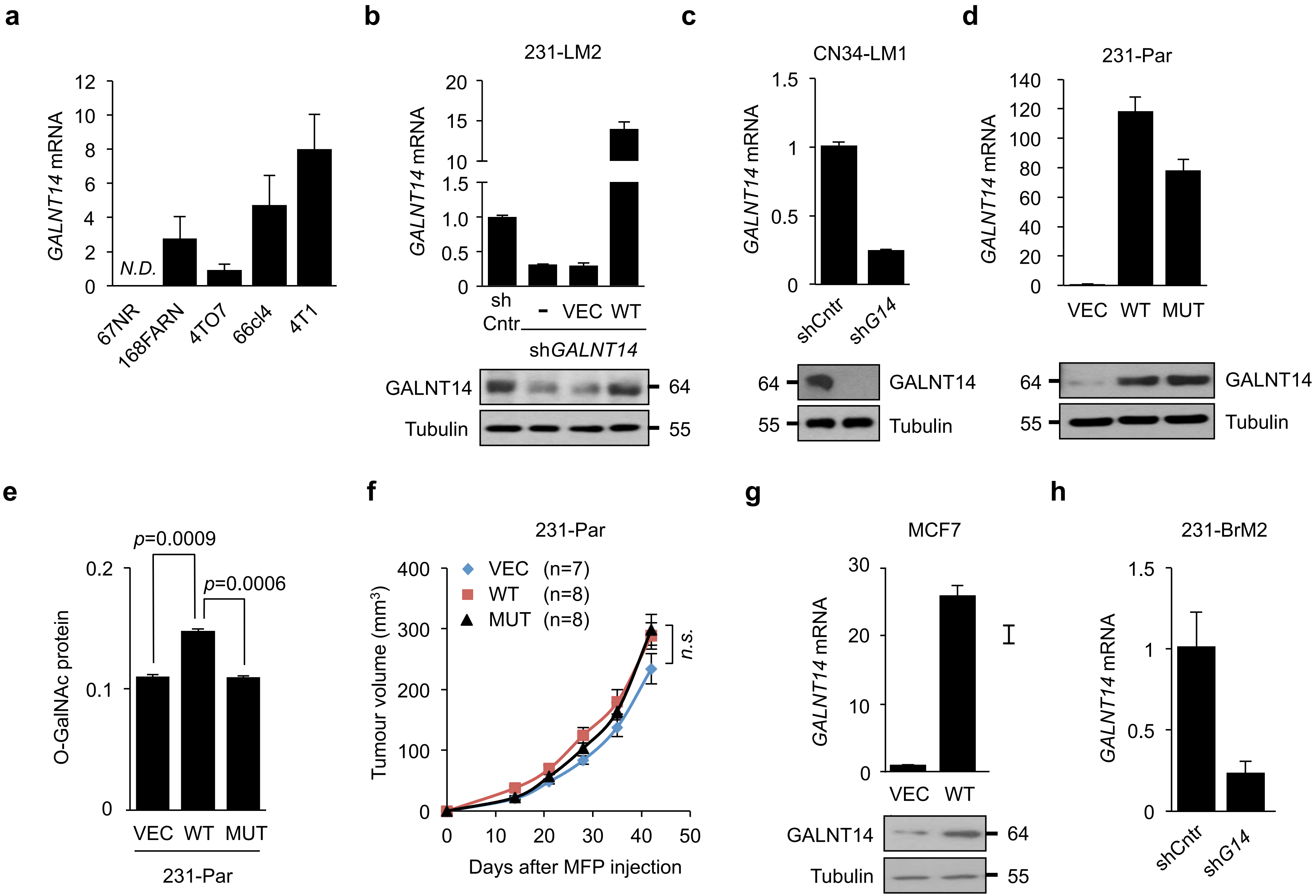

a
GALNT14 mRNA
N.D.
67NR
168FARN
4TO7
66cl4
4T1
b
231-LM2
GALNT14 mRNA
sh Cntr
-
VEC
WT
shGALNT14
GALNT14
Tubulin
64
55
c
CN34-LM1
GALNT14 mRNA
shCntr
shG14
GALNT14
Tubulin
d
231-Par
GALNT14 mRNA
VEC
WT
MUT
GALNT14
Tubulin
e
p=0.0009
p=0.0006
O-GalNAc protein
VEC
WT
MUT
231-Par
f
231-Par
VEC (n=7)
WT (n=8)
MUT (n=8)
Tumour volume (mm3)
n.s.
Days after MFP injection
g
MCF7
GALNT14 mRNA
VEC
WT
GALNT14
Tubulin
h
231-BrM2
GALNT14 mRNA
shCntr
shG14

Supplementary Figure 2 GALNT14 selectively promotes lung metastasis.

(**a**) Relative *GALNT14* mRNA levels in mouse breast cancer cells with varying lung metastasis activities. 67NR, non-metastatic; 168FARN and 4T07, weakly metastatic to lymph nodes; 66cl4, weakly lung metastatic; 4T1, highly metastatic to the lung and bones. *N.D.*: not detected.

(**b**) Relative *GALNT14* mRNA (top) or protein (bottom) levels in control, *GALNT14*-silenced, and rescued 231-LM2 cells. shCntr: non-targeting control shRNA, sh*GALNT14*: shRNA against *GALNT14*, VEC: control vector for a *GALNT14* expression vector, WT: wild-type *GALNT14* expression vector.

(**c**) Similar experiments as in **b** except that CN34-LM1 cells are used. sh*G14* indicates sh*GALNT14.*

(**d,e**) Relative mRNA (top) and protein (bottom) of GALNT14 **(d)** and O-GalNAc-glycosylated protein levels **(e)** in 231-Par cells expressing control vector (VEC), wild-type (WT) and catalytic mutant (MUT) GALNT14.

(**f**) The indicated 231-Par cells ($5x10^5$) were implanted into the mammary fat pads and tumour growth rates were measured.

(**g**) Relative *GALNT14* mRNA (top) and protein levels (bottom) of MCF7 cells expressing control vector or wild-type GALNT14 were analyzed.

(**h**) Relative expression of *GALNT14* in control and *GALNT14*-silenced 231-BrM2 cells.

P-values were calculated using two-tailed unpaired Student's t test except (f) using one-tailed Mann-Whitney test. Data are mean ± SEM (error bars). Western blot results in **b**,**c**,**d** and **g** are representative and were replicated at least two times. qRT-PCR data are representative of two independent experiments, each with triplicate samples.

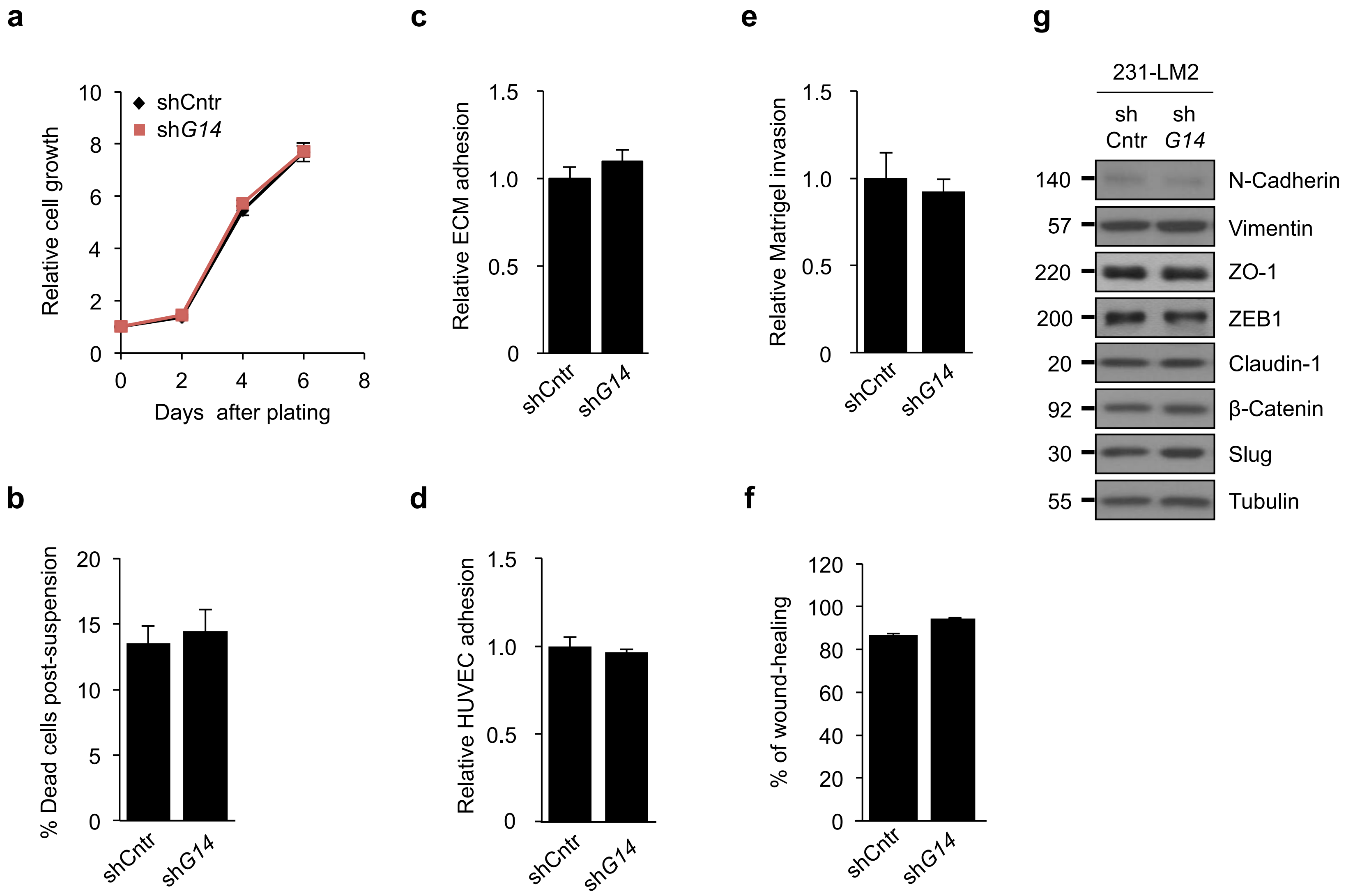

a
b
c
d
e
f
g
shCntr
shG14
Relative cell growth
Days after plating
% Dead cells post-suspension
Relative ECM adhesion
Relative HUVEC adhesion
Relative Matrigel invasion
% of wound-healing
231-LM2
sh Cntr
sh G14
140
57
220
200
20
92
30
55
N-Cadherin
Vimentin
ZO-1
ZEB1
Claudin-1
β-Catenin
Slug
Tubulin

Supplementary Figure 3 GALNT14 is not required for cell prolifertion, resistance to anoikis, motility or EMT.

(**a**) Comparison of growth rates between control and *GALNT14*-knockdown (sh*G14*) 231-LM2 cells. *n*=6.

(**b**) Relative anoikis resistance of the indicated 231-LM2 cells. Cells were grown under suspension conditions for 3 days in media containing 0.5% FBS and the number of dead or viable cells were counted by using trypan blue dye. The percentages of dead cells are shown. *n*=4.

(**c,d**) Comparative adhesion abilities of the indicated 231-LM2 cells to extra cellular matrix (ECM) or Human umbilical vein endothelial cell (HUVEC). Cells were seeded onto the plates coated with matrigel (**c**) or HUVEC monolayer (**d**) and numbers of adhered cancer cells were assessed by measuring the luminescent signals. *n*=6.

(**e**) Invasion abilities of the indicated 231-LM2 cells. Cells were labeled with cell tracker dye and plated onto matrigel-coated trans-well inserts. Images of migrating cells were captured at X10 magnification and quantified with Image J software. *n*=6.

(**f**) Relative migration of the indicated 231-LM2 cells. Wound healing motility assays were performed measuring wound closure following by scratching the cell monolayer. Images of migrating cells were captured at X4 magnification. *n*=9.

(**g**) Analysis of EMT markers in the indicated 231-LM2 cells by western blotting analysis.
Data are mean ± SEM (error bars).

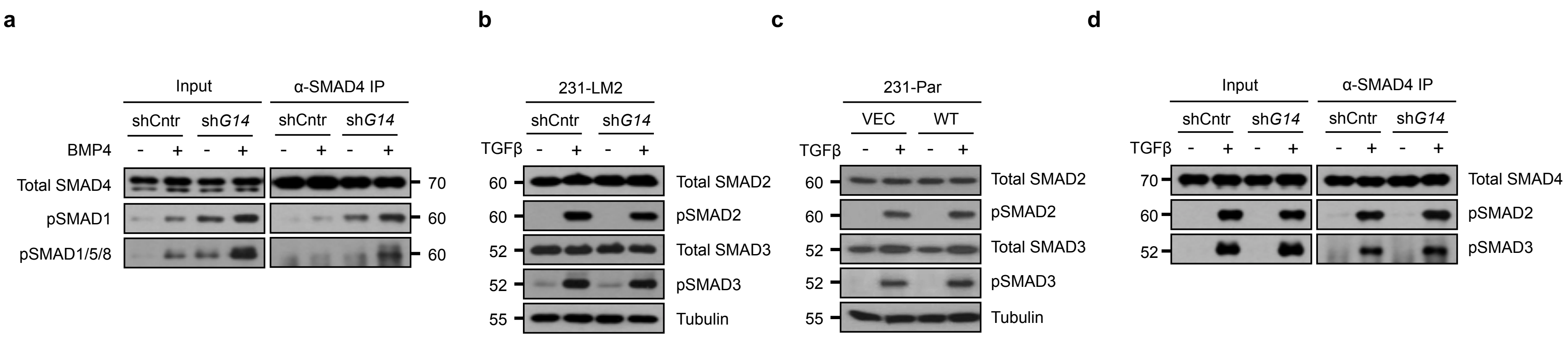

Supplementary Figure 4 Analysis of BMP and TGF signaling pathways upon changes in *GALNT14* expression. (**a**) Control and sh*G14*-expressing 231-LM2 cells were treated with vehicle or BMP4 (10 ng ml^{-1} for 15 minutes). Nuclear extract was isolated and subjected to immuno-precipitation with an antibody against SMAD4 and co-immunoprecipitated pSMAD1 and pSMAD1/5/8 were analyzed by immunoblotting.

(**b,c**) The indicated 231-LM2 (**b**) and 231-Par (**c**) cells were treated with TGFβ (100 pM for 15 minutes) and total and phosphorylated levels of the indicated SMADs were analyzed by western blotting.

(**d**) Similar experiments as in **a** except that interaction between pSMAD 2/3 and SMAD4 was analyzed in the presence of TGFβ (100 pM for 15 minutes).

Western blot images are representative of three independent experiments.

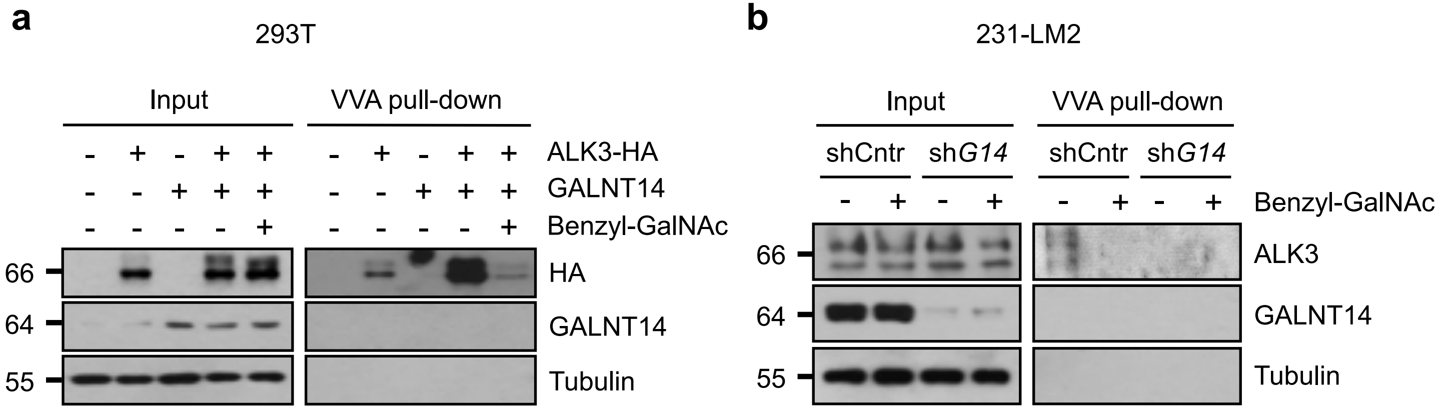

Supplementary Figure 5 BMPR 1A (ALK3) is O-GalNAcylated by GALNT14.

(**a**) Pull-down assays with VVA-agarose were performed as shown in Fig. 4f using 293T cells transfected with the indicated construct. 1mM benzyl-GalNAc (BG) was added to the media 24 hours prior to IP in lane 5.

(**b**) Similar experiments as in **a** except that O-GalNAcylation of endogenous ALK3 in shCntr and sh*G14*-expressing 231-LM2 cells were analysed.

Western blot images are representative of three independent experiments.

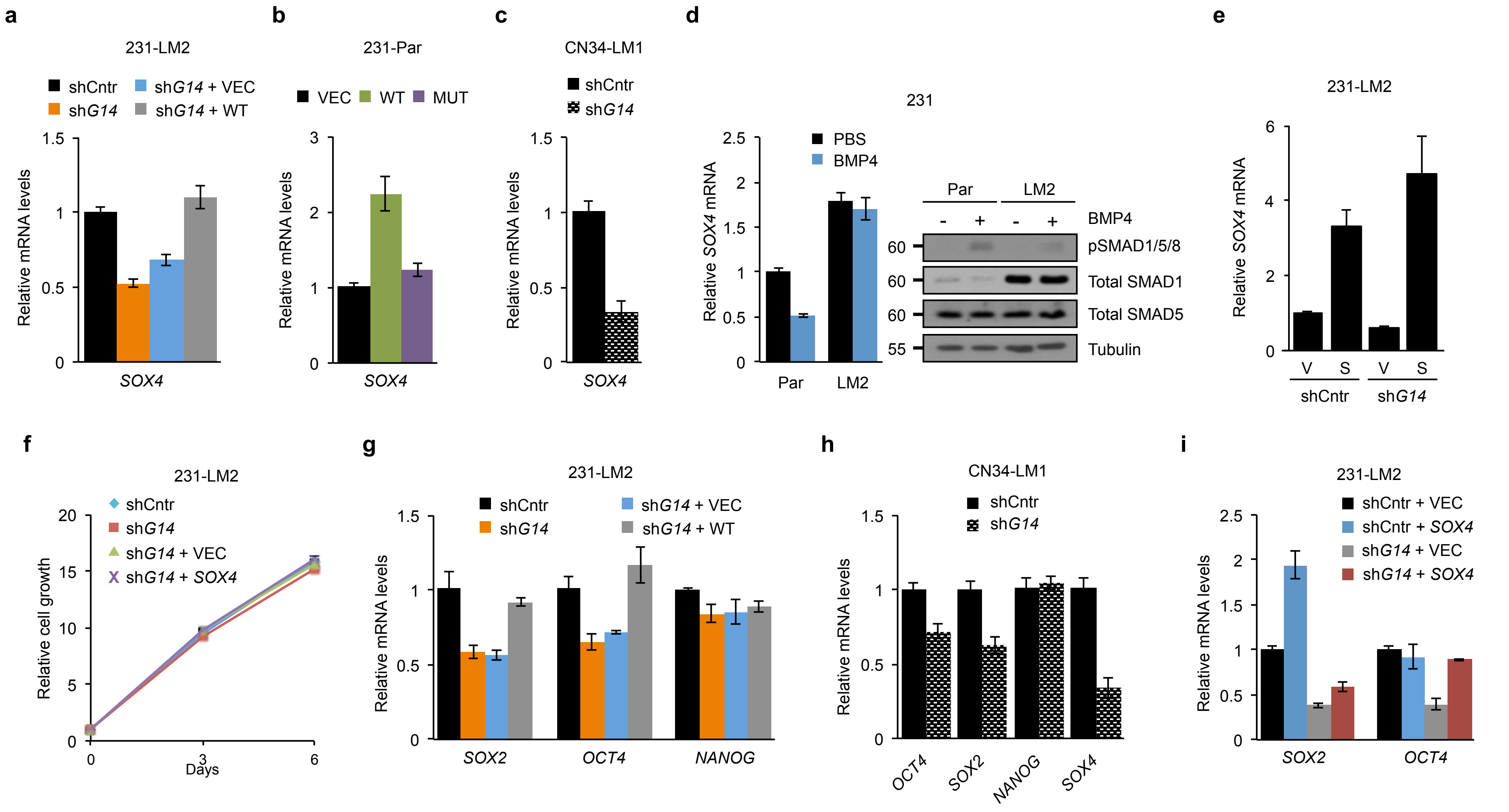

a
231-LM2
shCntr
shG14
shG14 + VEC
shG14 + WT
Relative mRNA levels
SOX4
b
231-Par
VEC
WT
MUT
Relative mRNA levels
SOX4
c
CN34-LM1
shCntr
shG14
Relative mRNA levels
SOX4
d
231
PBS
BMP4
Relative SOX4 mRNA
Par
LM2
BMP4
pSMAD1/5/8
Total SMAD1
Total SMAD5
Tubulin
e
231-LM2
Relative SOX4 mRNA
V
S
shCntr
shG14
f
231-LM2
shCntr
shG14
shG14 + VEC
shG14 + SOX4
Relative cell growth
Days
g
231-LM2
shCntr
shG14
shG14 + VEC
shG14 + WT
Relative mRNA levels
SOX2
OCT4
NANOG
h
CN34-LM1
shCntr
shG14
Relative mRNA levels
OCT4
SOX2
NANOG
SOX4
i
231-LM2
shCntr + VEC
shCntr + SOX4
shG14 + VEC
shG14 + SOX4
Relative mRNA levels
SOX2
OCT4

Supplementary Figure 6 GALNT14 alleviates the BMP-mediated suppression of *SOX4* expression.

(**a-c**) Relative expression of *SOX4* in the indicated 231-LM2 (**a**), 231-Par (**b**) and CN34 (**c**) cells. sh*G14* indicates sh*GALNT14. n*=6.

(**d**) BMP-responsiveness of 231-Par and LM2 cells. Relative *SOX4* mRNA levels upon the addition of 10 ng ml^{-1} of BMP4 (left). Cell lysates were subjected to immunoblotting analyses with antibodies against the indicated proteins (right). *n*=6.

(**e**) Relative *SOX4* mRNA levels in the indicated 231-LM2 cells. V: control vector, S*: SOX4* expression vector. *n*=6.

(**f**) Comparative cell growth of the indicated 231-LM2 cells. *n*=6.

(**g,h**) Relative expression levels of *SOX4, SOX*2, *OCT4*, and *NANOG* in the indicated 231-LM2 (**g**) and CN34-LM1 (**h**) cells. *n*=6.

(i) Relative *SOX4* expression levels in the indicated 231-LM2 cells. *n*=6.

Data are mean ± SEM (error bars). Western blot images in **d** are representatives of two independent experiments.

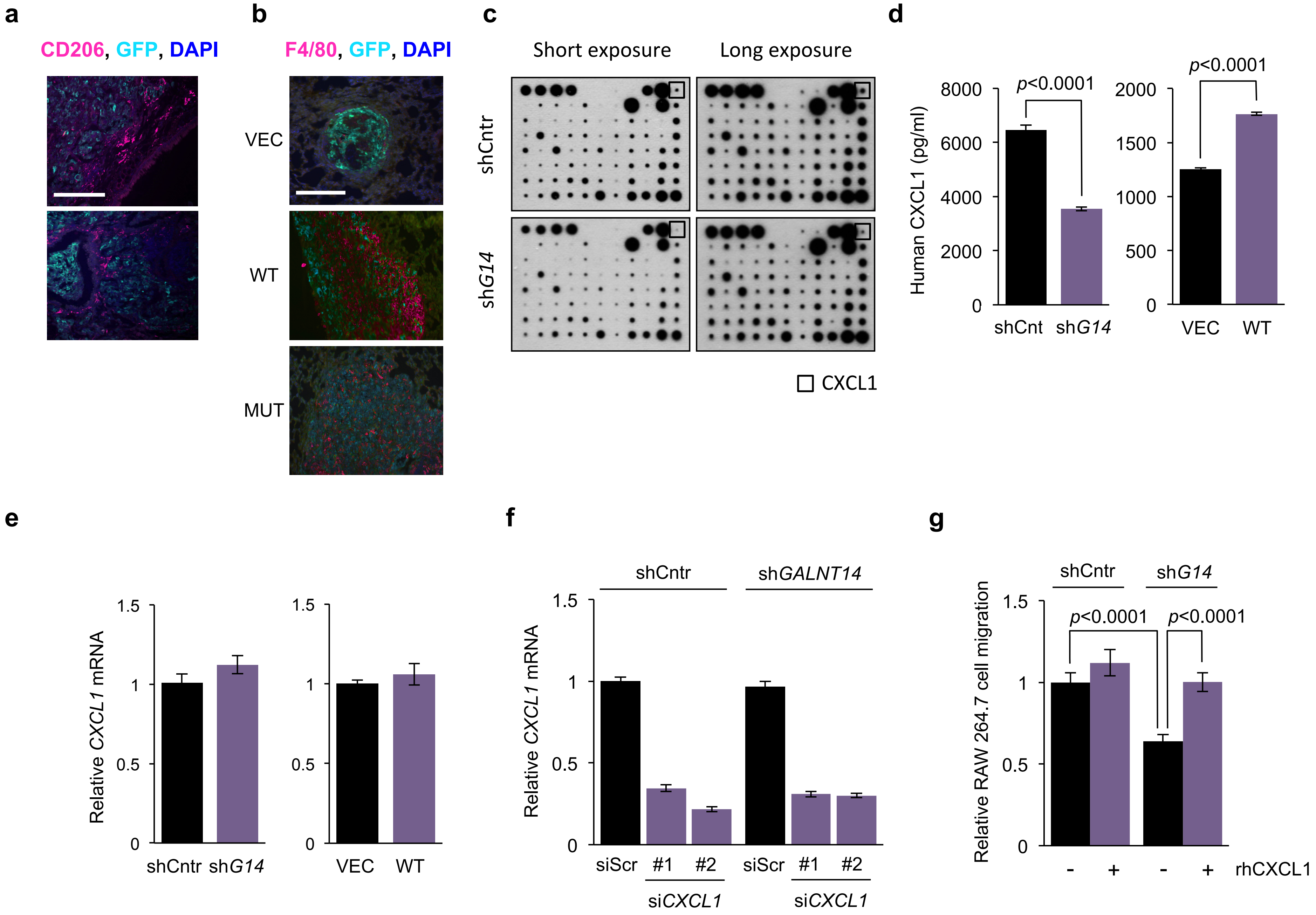

a
CD206, GFP, DAPI
b
F4/80, GFP, DAPI
VEC
WT
MUT
c
Short exposure
Long exposure
shCntr
shG14
CXCL1
d
Human CXCL1 (pg/ml)
p<0.0001
shCnt
shG14
VEC
WT
e
Relative CXCL1 mRNA
shCntr
shG14
VEC
WT
f
shCntr
shGALNT14
Relative CXCL1 mRNA
siScr
#1
#2
siCXCL1
g
shCntr
shG14
Relative RAW 264.7 cell migration
p<0.0001
rhCXCL1

Supplementary Figure 7 GALNT14 endows BCCs with an ability to modify the lung microenvironment.

(**a**) CD206 immunofluorescent staining of frozen lung sections from mice injected with the 231-LM2 expressing shCntr. Two representative areas of lung nodules are shown. GFP-positive cells represent cancer cells. Scale bar, 100 μm.

(b) F4/80 Representative immunofluorescent staining of frozen lung sections from mice injected with the indicated 231-Par cells. Representative images are shown. GFP-positive cells represent cancer cells. Scale bar, 100 μm.

(**c**) Conditioned media collected from shCntr or sh*G14*-expressing 231-LM2 cells were subjected to cytokine array (Ray Biotech).

(**d**) Conditioned media (CM) from the indicated 231-LM2 (left) and 231-Par (right) cells were subjected to CXCL1 ELISA. *n*=6.

(**e**) Relative *CXCL1* mRNA levels in the indicated 231-LM2 (left) and 231-Par (right) cells. *n*=6.

(**f**) The sh*Cntr* or sh*GALNT14*-expressing 231-LM2 cells were transfected with two independent siRNAs against *CXCL1.* Relative expression levels of *CXCL1* were analyzed by qRT-PCR. *n*=6.

(**g**) RAW 264.7 cell transwell migration assay as in Fig. 6c except that recombinant human CXCL1 (5 ng ml^{-1}) was added to CM from the indicated 231-LM2 cells. *n*=9.

P-values were calculated using two-tailed unpaired Student's t test. Data are mean ± SEM (error bars).

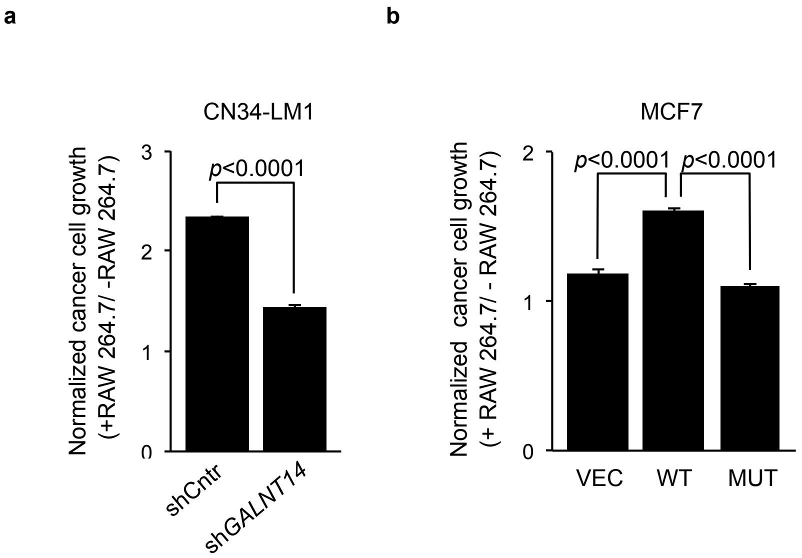

Supplementary Figure 8 GALNT14 enhances macrophage-stimulated growth of BCCs.

Relative growth of the indicated CN34-LM1 (**a**) and MCF7 (**b**) cells in the absence or presence of RAW 264.7 macrophage cells. *n*=6.

P-values were calculated using two-tailed unpaired Student's t test. Data are mean ± SEM (error bars).

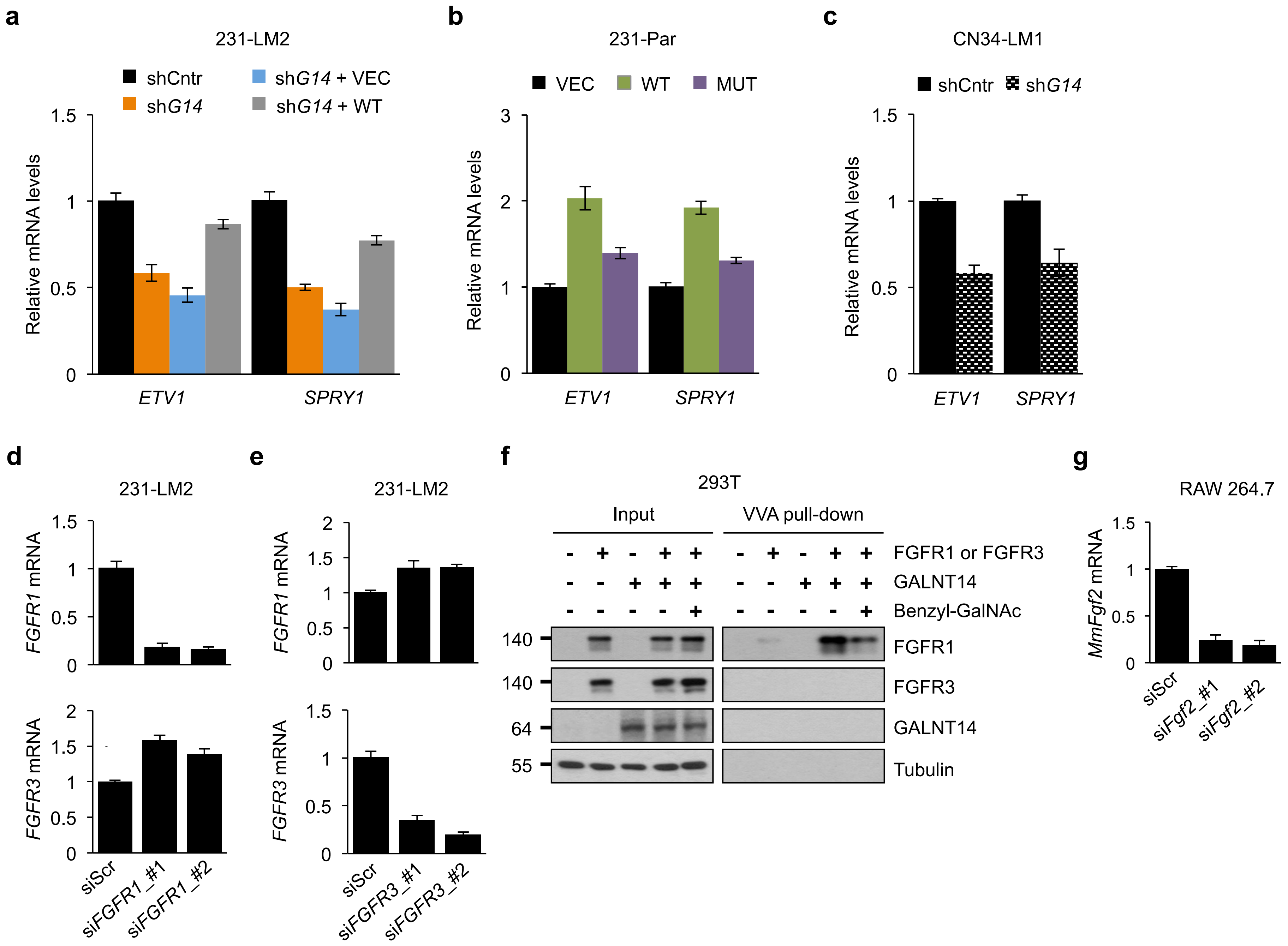
a
231-LM2
shCntr
shG14
shG14 + VEC
shG14 + WT
Relative mRNA levels
ETV1
SPRY1
b
231-Par
VEC
WT
MUT
Relative mRNA levels
ETV1
SPRY1
c
CN34-LM1
shCntr
shG14
Relative mRNA levels
ETV1
SPRY1
d
231-LM2
FGFR1 mRNA
FGFR3 mRNA
siScr
siFGFR1_#1
siFGFR1_#2
e
231-LM2
FGFR1 mRNA
FGFR3 mRNA
siScr
siFGFR3_#1
siFGFR3_#2
f
293T
Input
VVA pull-down
FGFR1 or FGFR3
GALNT14
Benzyl-GalNAc
140
140
64
55
FGFR1
FGFR3
GALNT14
Tubulin
g
RAW 264.7
MmFgf2 mRNA
siScr
siFgf2_#1
siFgf2_#2

Supplementary Figure 9 GALNT14 activates FGF signaling in BCCs.
(**a-c**) Relative mRNA levels of FGF-responsive genes *ETV1* and *SPRY1* in the indicated 231-LM2 (**a**), 231-Par (**b**) and CN34-LM1 (**c**) cells. *n*=6.

(**d**) Relative mRNA levels of *FGFR1* (top) and *FGFR3* (bottom) in 231-LM2, transfected with scrambled (siScr) or two independent siRNAs against *FGFR1*. *n*=6.

(**e**) Similar experiment as in **d** except that siRNAs against *FGFR3* were used. *n*=6.

(**f**) O-glycosylation of FGF receptors. Similar experiments as in Fig. 4f except that HEK 293T cells were transfected with *FGFR1* or *FGFR3* expression vectors. 1 mM benzyl-GalNAc was added to the media 24 hours prior to IP where indicated. Western blot images are representative of three independent experiments.

(**g**) Relative expression of *MmFgf2* in RAW264.7 cells transfected with two independent siRNAs against this gene. *n*=6.

Data are mean ± SEM (error bars).

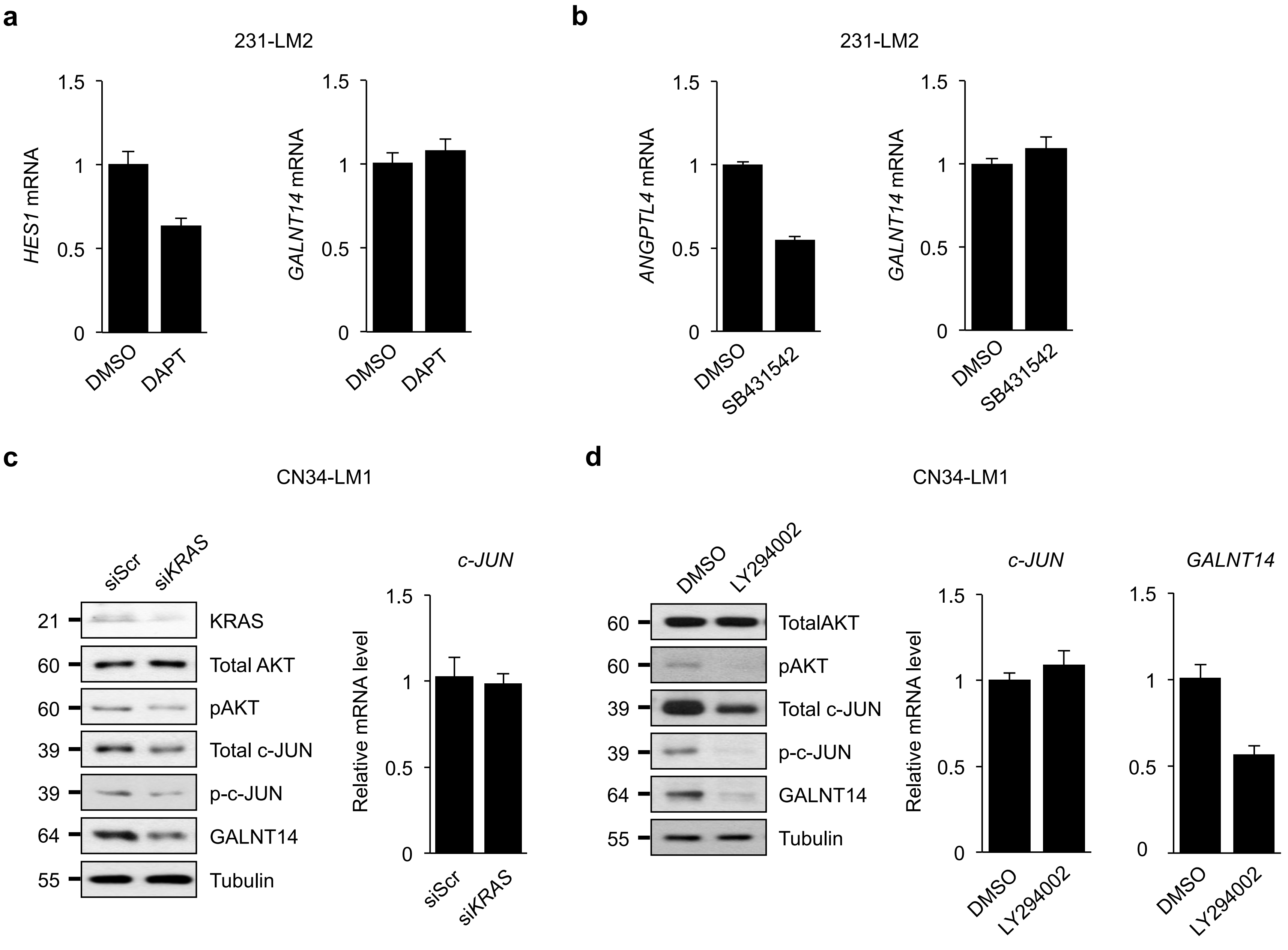
a
231-LM2
HES1 mRNA
GALNT14 mRNA
1.5
1
0.5
0
DMSO
DAPT
b
231-LM2
ANGPTL4 mRNA
GALNT14 mRNA
DMSO
SB431542
c
CN34-LM1
siScr
siKRAS
21
60
39
64
55
KRAS
Total AKT
pAKT
Total c-JUN
p-c-JUN
GALNT14
Tubulin
c-JUN
Relative mRNA level
d
CN34-LM1
DMSO
LY294002
TotalAKT
pAKT
Total c-JUN
p-c-JUN
GALNT14
Tubulin
c-JUN
GALNT14
Relative mRNA level

Supplementary Figure 10 Activated KRAS-PI3K-c-JUN, but not NOTCH or TGFβ signaling pathways, increases *GALNT14* transcription in lung metastatic BCCs.

(**a,b**) Relative *GALNT14* mRNA levels in the presence of NOTCH inhibitor DAPT (5 μM) (**a**) and TGFβ inhibitor SB431542 (30 μM) (**b**). *HES1* and *Angiopoietin-like 4* (*ANGPTL4*) were used as a positive control for DAPT and SB431542, respectively. *n*=6.

(**c**) Changes in total and phosphorylated c-JUN and AKT and GALNT14 protein (left) and *c-JUN* mRNA (right) levels in CN34-LM1 cells upon *KRAS* knockdown. *n*=6.

(**d**) Left: Comparison of total and phosphorylated c-JUN and AKT as well as GALNT14 protein level in the absence or presence of LY294002 (30 μM). Right: *GALNT14* and *c-JUN* mRNA levels under the same condition. *n*=6.

Data are mean ± SEM (error bars). Western blot images in **c** and **d** are representative and were replicated at least two times.

Fig. 2a

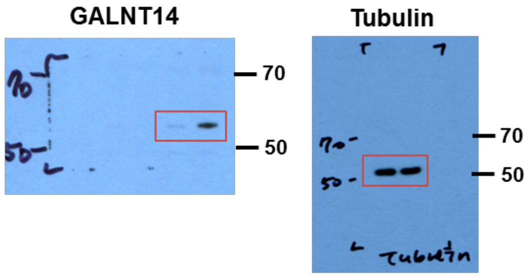

Fig. 4d

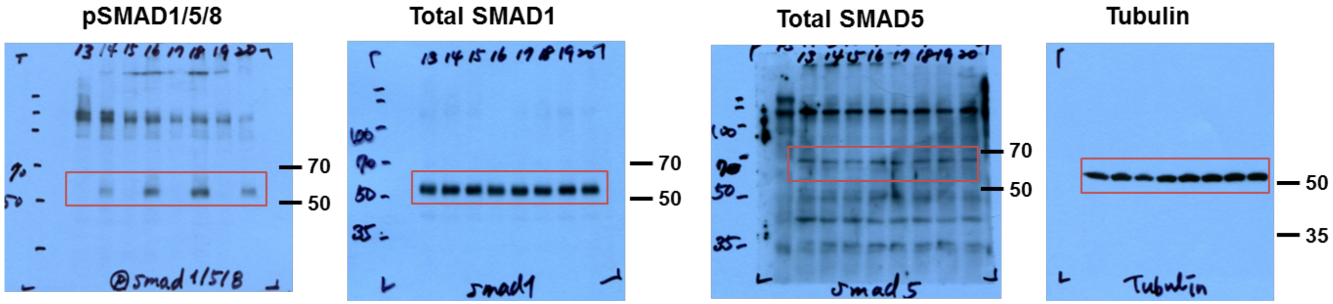

Supplementary Figure 11 Unprocessed scans of western blots.

Fig. 4e

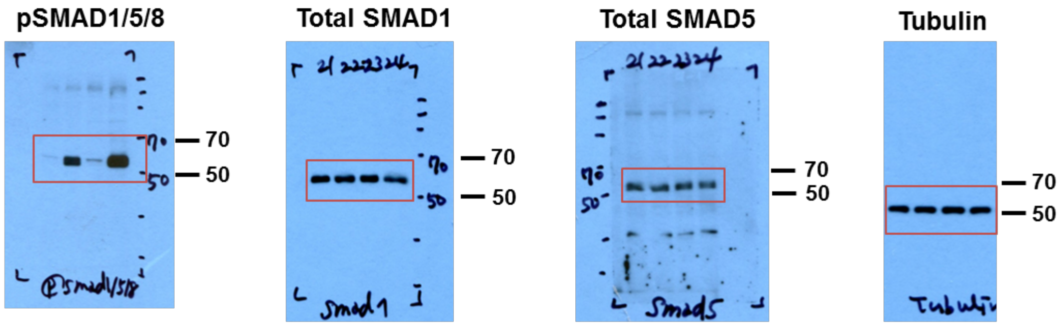

Fig. 4f

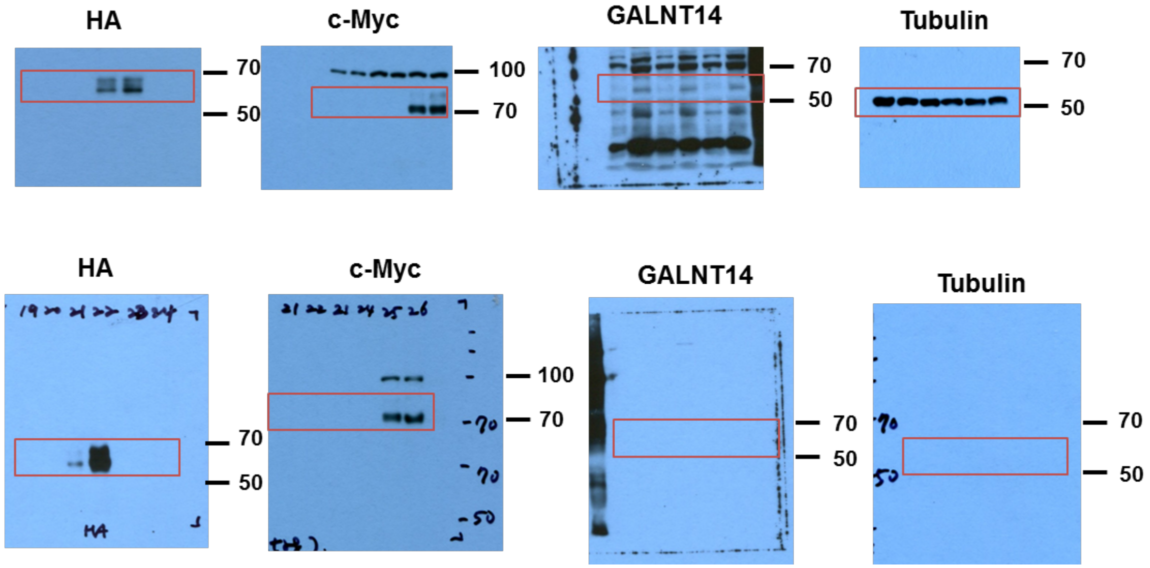

Supplementary Figure 11 Unprocessed scans of western blots. (Continued)

Fig. 6d

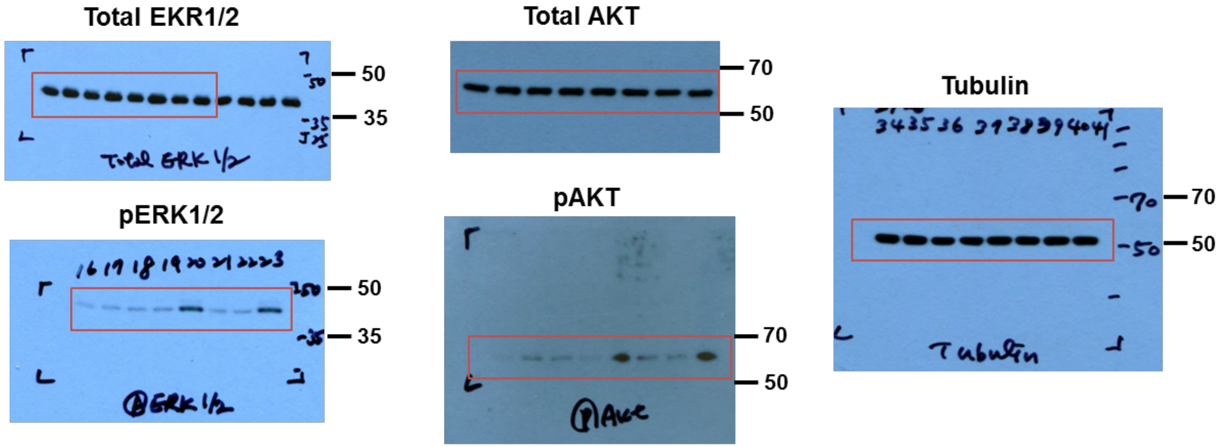

Fig. 7c

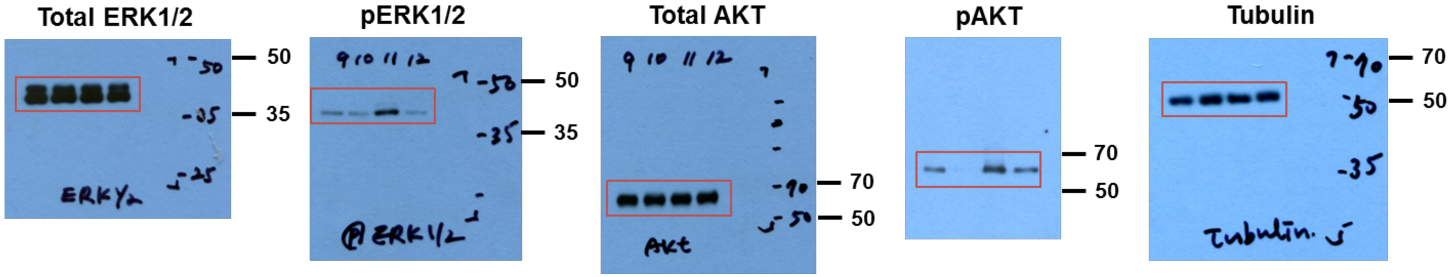

Supplementary Figure 11 Unprocessed scans of western blots.

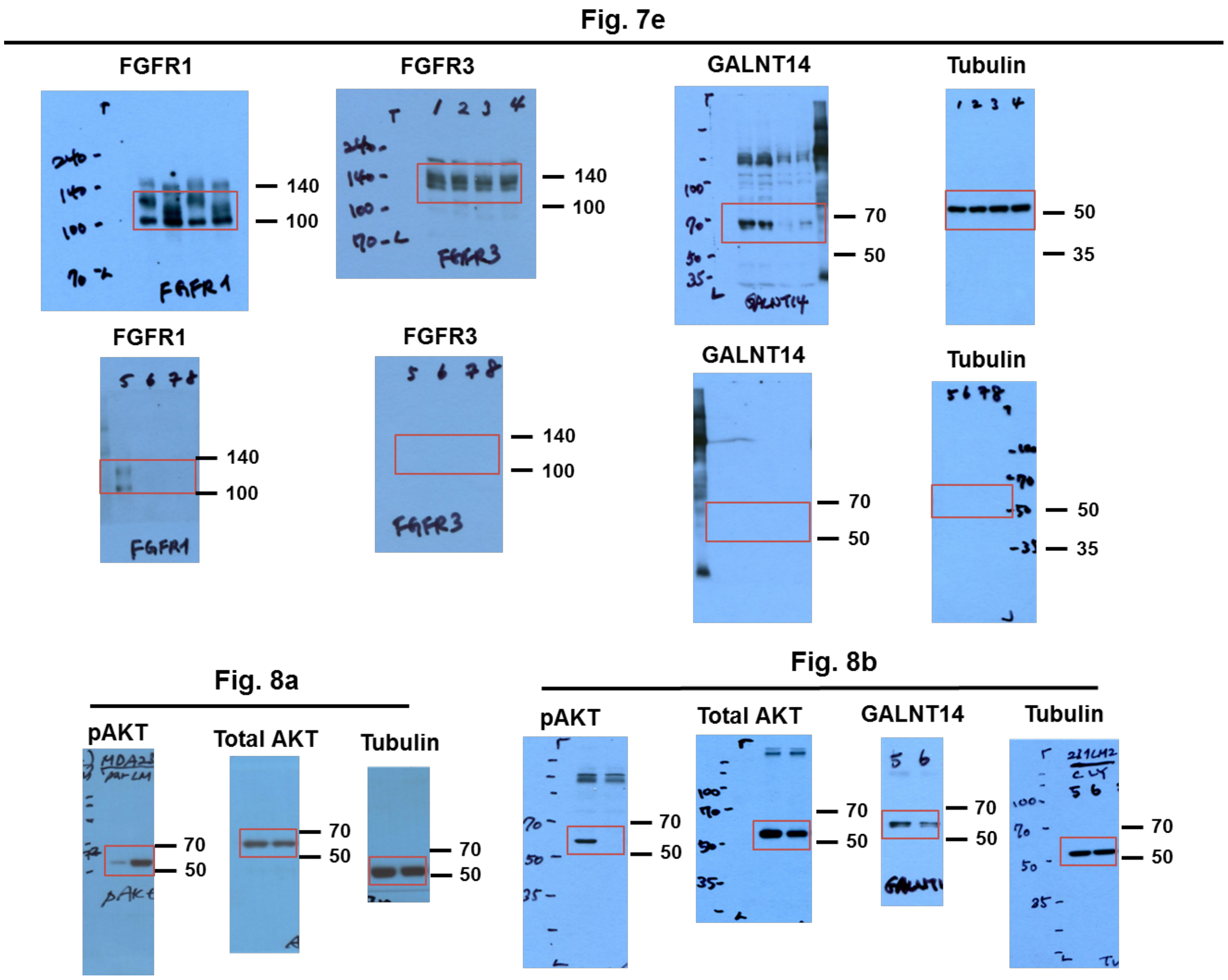

Supplementary Figure 11 Unprocessed scans of western blots. (Continued)

Fig. 8c

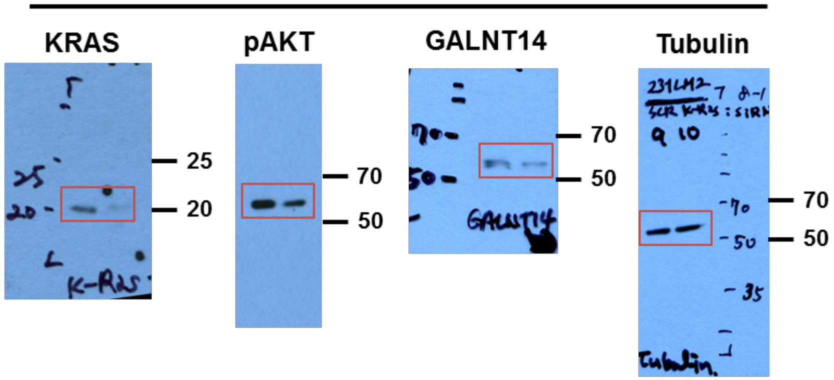

Fig. 8e

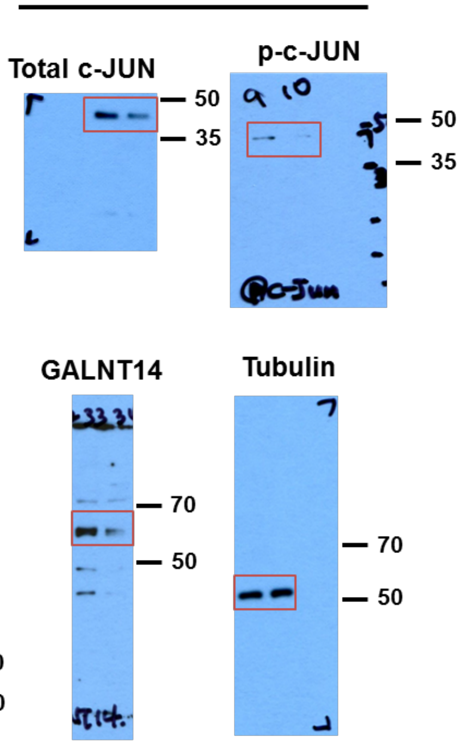

Fig. 8d

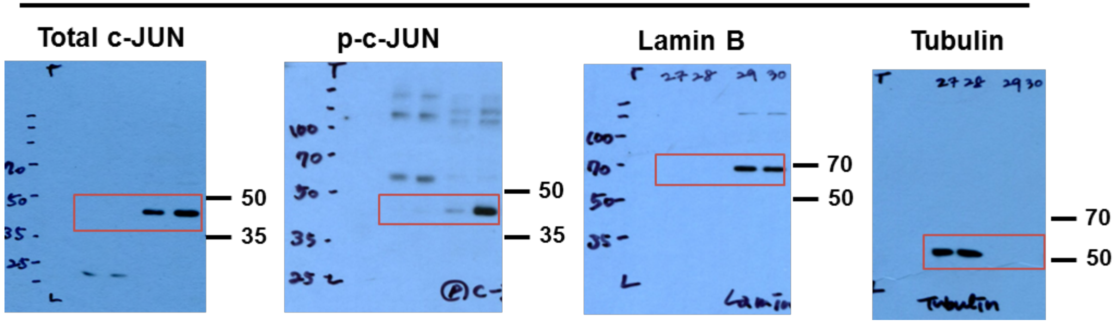

Supplementary Figure 11 Unprocessed scans of western blots. (Continued)

Fig. 8f

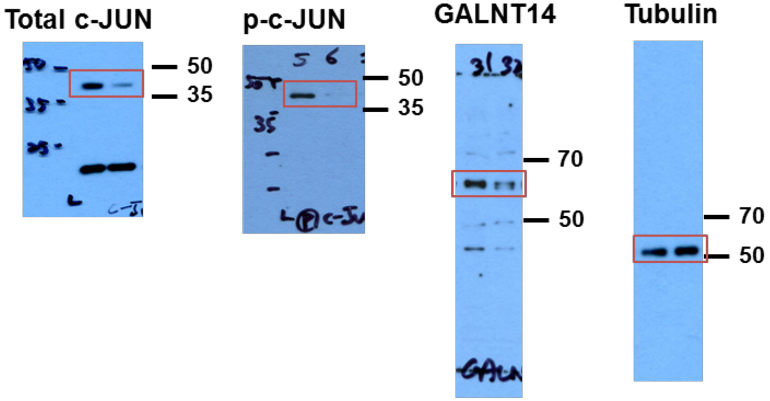

Fig. 8g

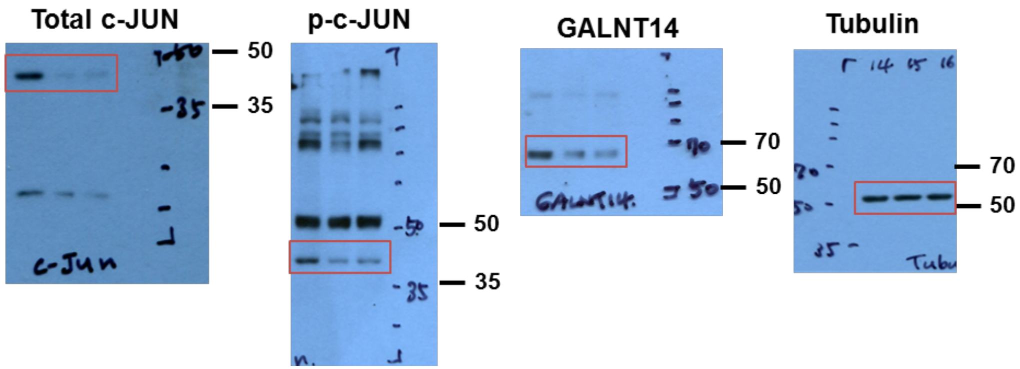

Fig. 8h

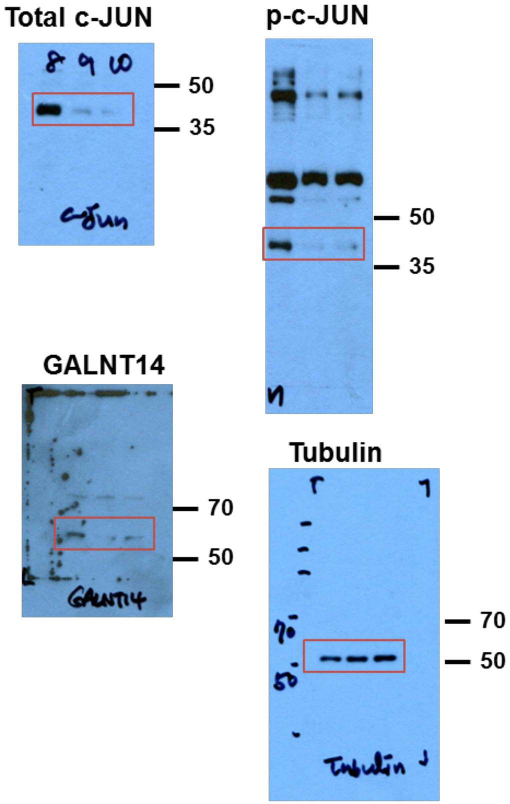

Supplementary Figure 11 Unprocessed scans of western blots. (Continued)

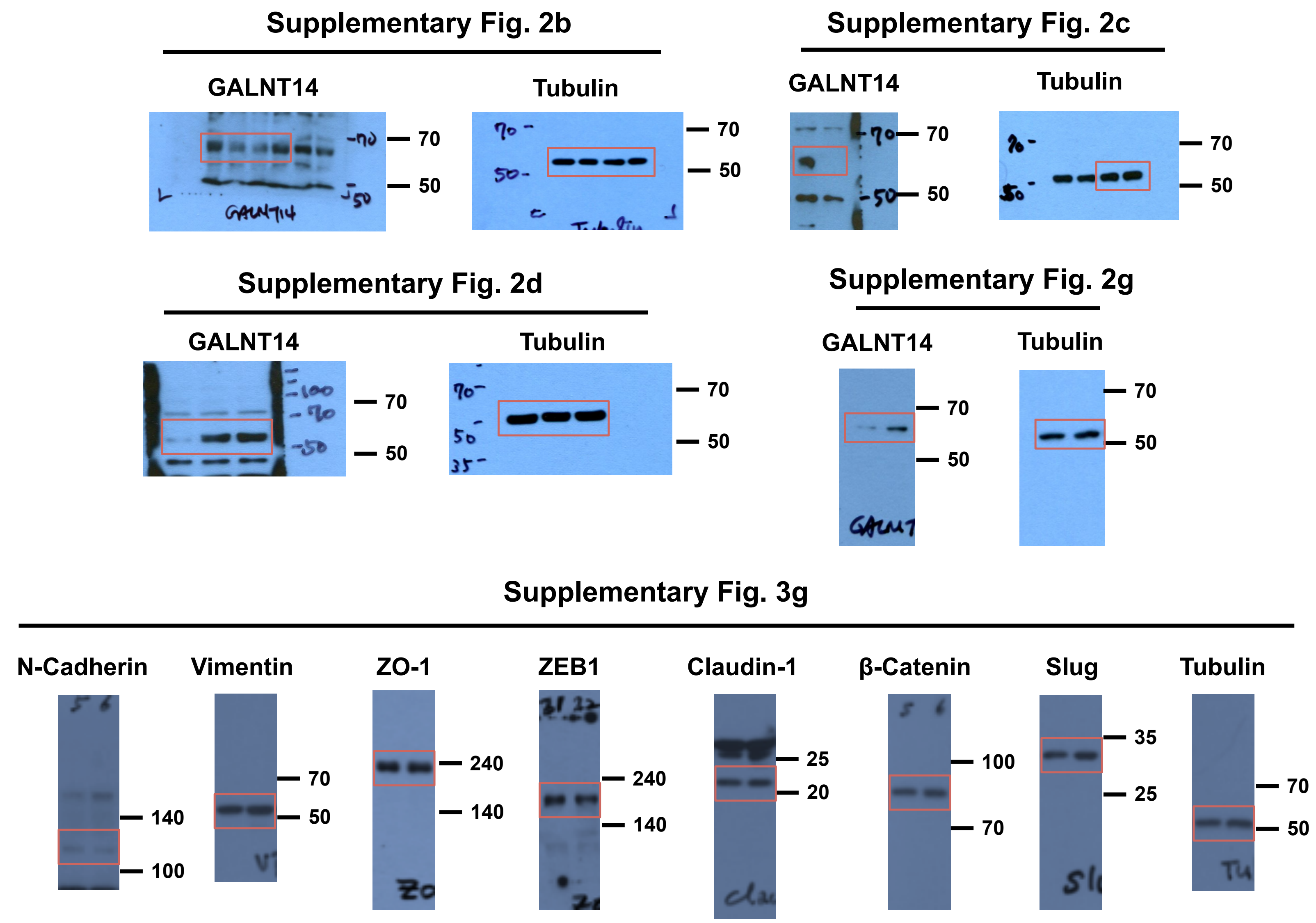

Supplementary Figure 11 Unprocessed scans of western blots. (Continued)

Supplementary Fig. 4a

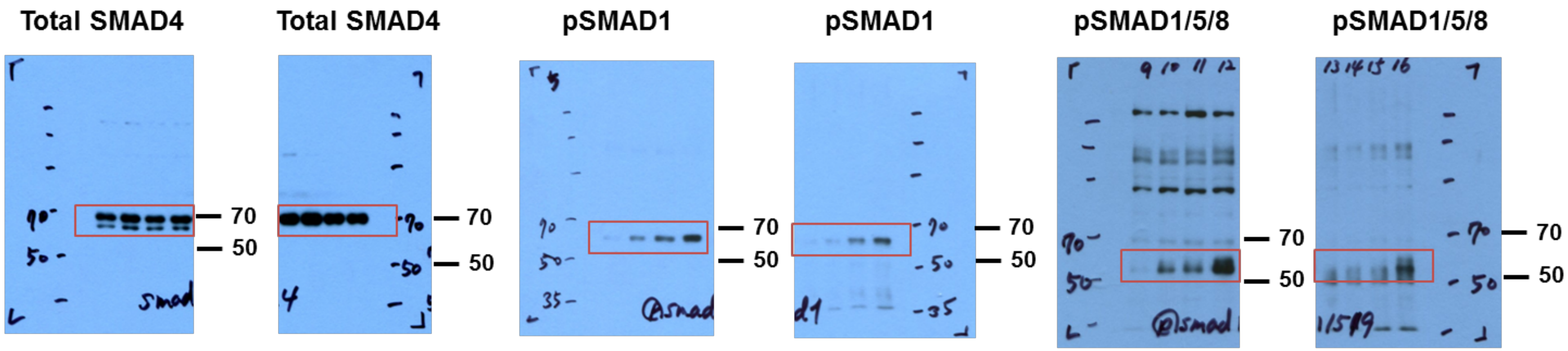

Supplementary Fig. 4b

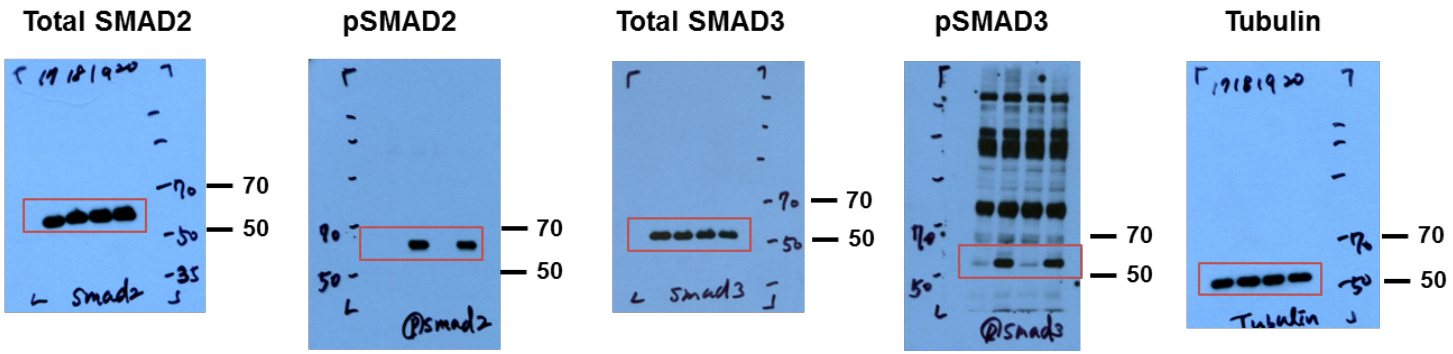

Supplementary Figure 11 Unprocessed scans of western blots. (Continued)

Supplementary Fig. 4c

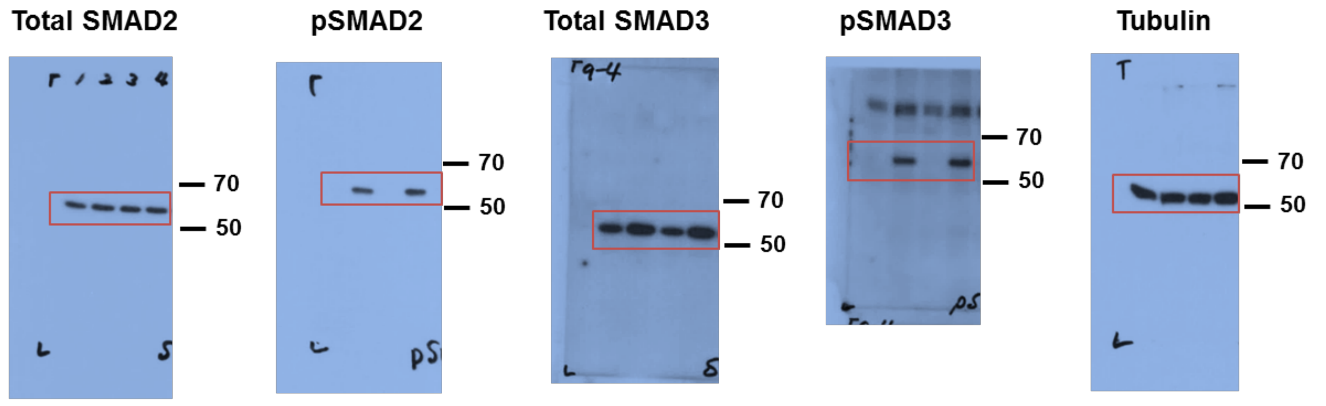

Supplementary Fig. 4d

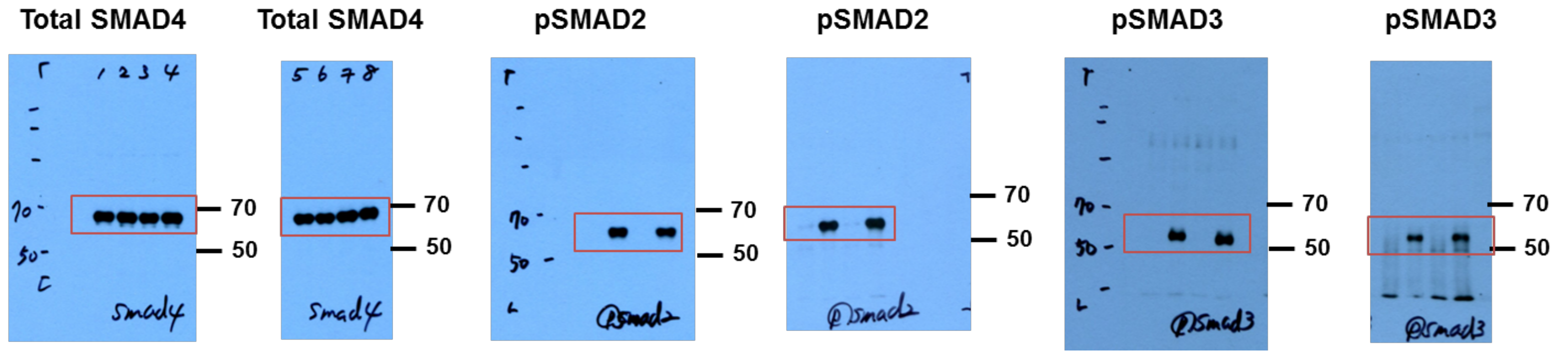

Supplementary Figure 11 Unprocessed scans of western blots. (Continued)

Supplementary Fig. 5a

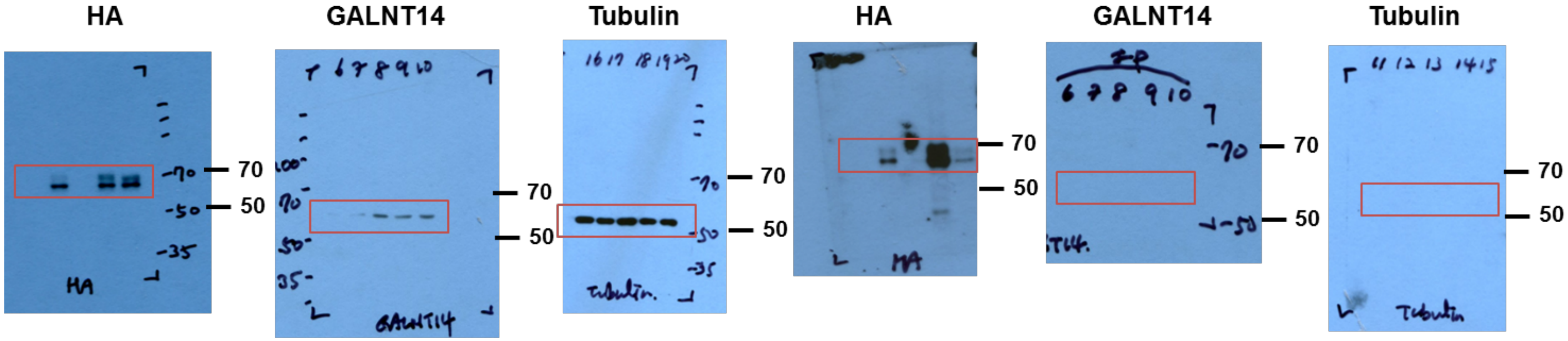

Supplementary Fig. 5b

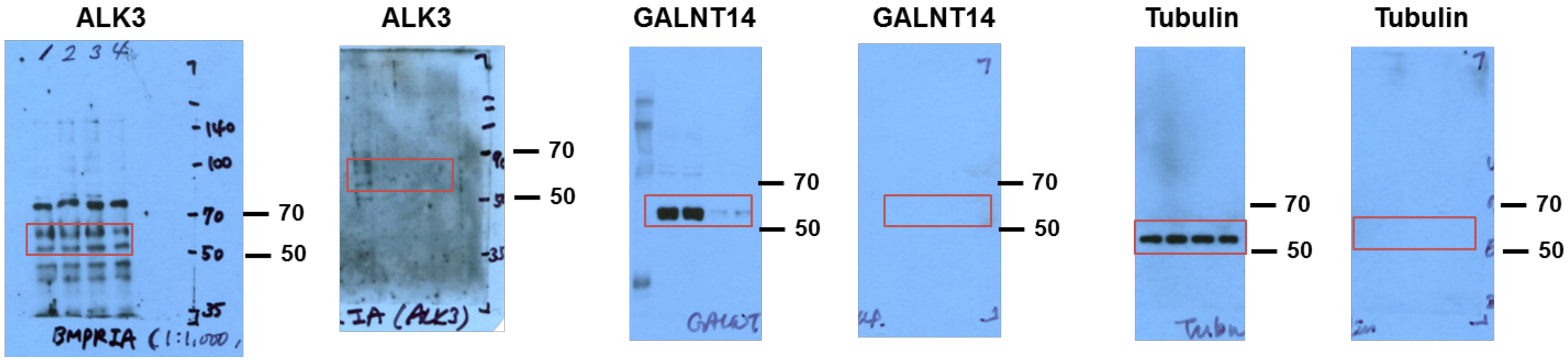

Supplementary Figure 11 Unprocessed scans of western blots. (Continued)

Supplementary Fig. 6d

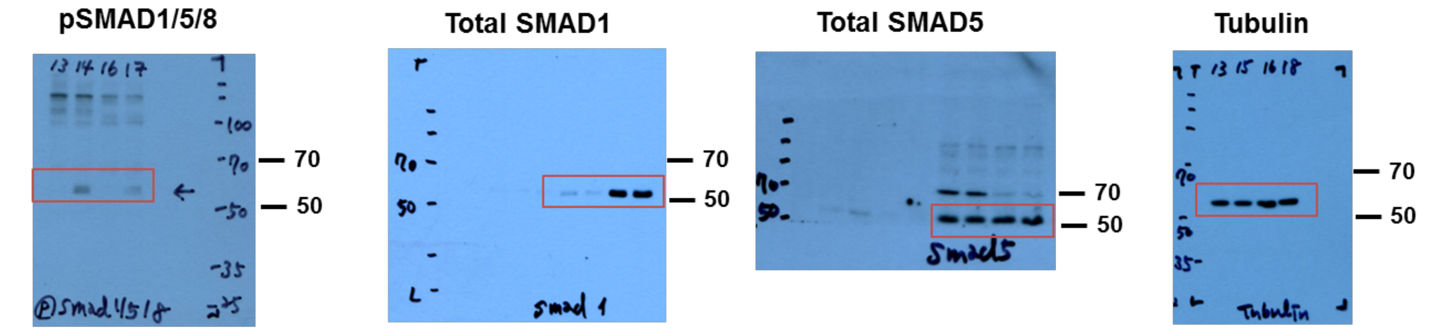

Fig. 9f

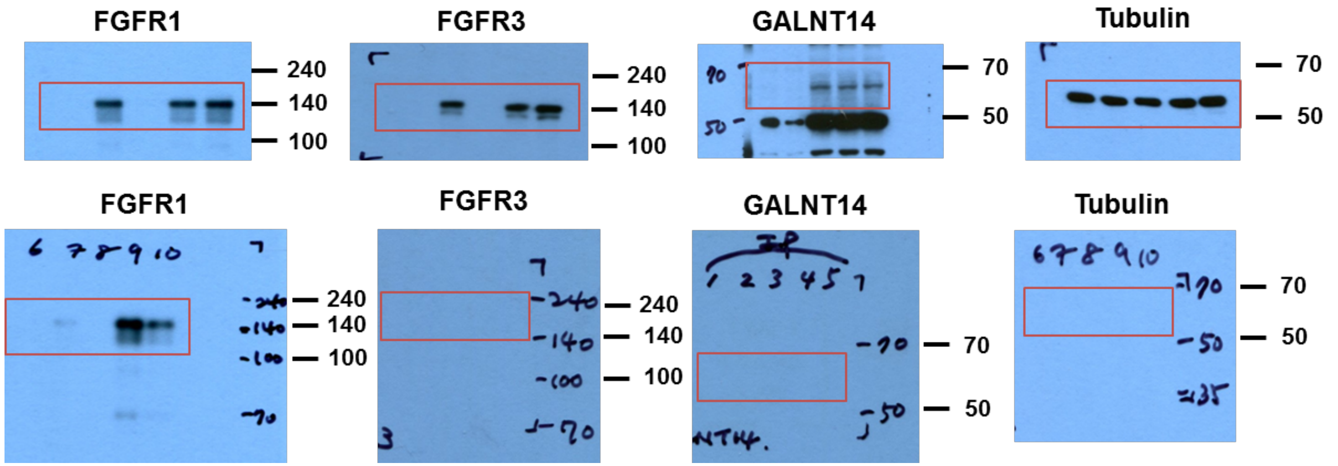

Supplementary Figure 11 Unprocessed scans of western blots. (Continued)

Supplementary Fig. 10c

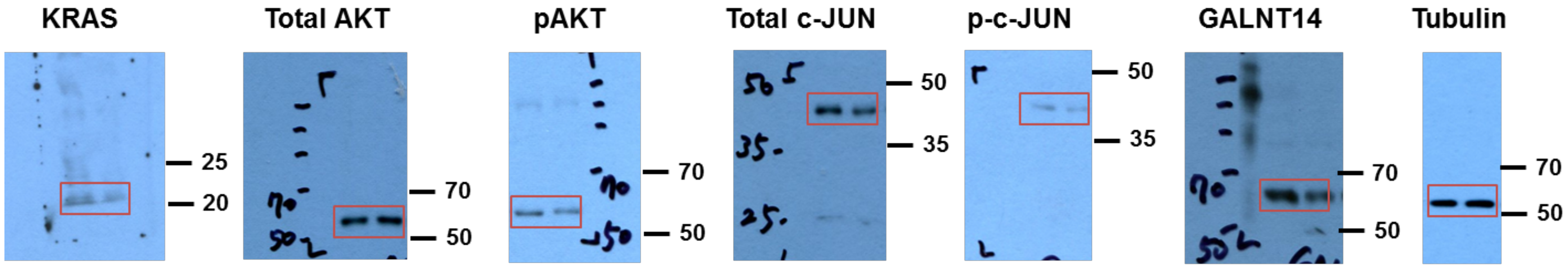

Supplementary Fig. 10d

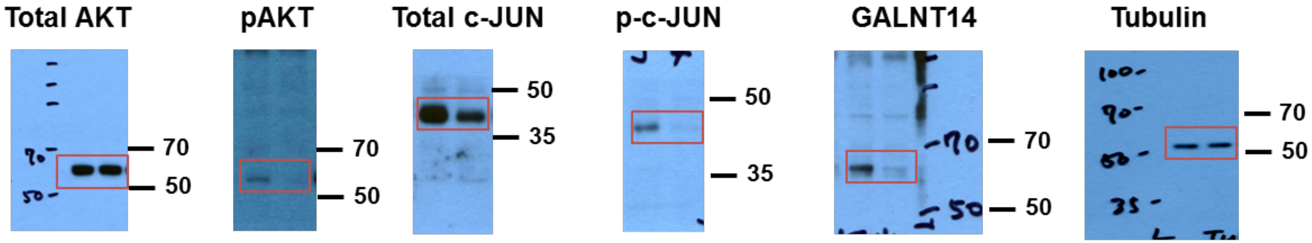

Supplementary Figure 11 Unprocessed scans of western blots. (Continued)

Supplementary Table 1 Recurrence rates according to *GALNT14* expression level in combined cohorts of EMC192 and MSK82, Related to Fig. 1c.

***GALNT14* Expression**	**Relapse Rate**		
	**Lung**	**Bone**	**Brain**
***GALNT14* Low (138)**	17/138 (12.3%)	59/138 (42.8%)	8/138 (5.8%)
***GALNT14* High (133)**	34/133 (25.6%)	57/133 (42.9%)	10/133 (7.5%)

Supplementary Table 2 A list of differentially expressed genes upon depletion or ectopic expression of *GALNT14*, Related to Fig. 5a.

17 up- and 13 down-regulated genes by GALNT 14 are listed. Fold-changes (shCntr/sh*GALNT14* and WT/VEC) are shown. The details are described in the text.

Gene Symbol	shCntr /sh*GALNT14*	WT/VEC	Gene Description
GALNT14	5.09	686.15	UDP-N-acetyl-alpha-D-galactosamine:polypeptide N-acetylgalactosaminyltransferase 14 (GalNAc-T14)
SOX4	3.27	4.04	SRY (sex determining region Y)-box 4
SLCO4A1	2.21	1.29	solute carrier organic anion transporter family, member 4A1
PTGES	1.83	1.39	prostaglandin E synthase
RP11-93B14.5	1.83	1.78	ribosomal protein L11
SPRY1	1.66	1.27	sprouty homolog 1, antagonist of FGF signaling (Drosophila)
MARCH2	1.55	1.35	membrane-associated ring finger (C3HC4) 2
KLF9	1.54	1.44	Kruppel-like factor 9
GGT5	1.52	1.74	gamma-glutamyltransferase 5
CDK15	1.47	1.32	PFTAIRE protein kinase 2
SAT1	1.47	1.31	spermidine/spermine N1-acetyltransferase 1
BDH2	1.42	1.43	3-hydroxybutyrate dehydrogenase, type 2
NR2F1-AS1	1.39	1.32	NR2F1 Antisense RNA
DNAJB5	1.39	1.28	DnaJ (Hsp40) homolog, subfamily B, member 5
ETV1 (ER81)	1.36	1.20	ets variant 1
TTC8	1.30	1.23	tetratricopeptide repeat domain 8
ARMCX2	1.28	1.30	armadillo repeat containing, X-linked 2
CLK4	1.27	1.22	CDC-like kinase 4
ATG101	0.72	0.78	autophagy related 101

CDC42EP2	0.70	0.84	CDC42 effector protein (Rho GTPase binding) 2
FJX1	0.72	0.81	four jointed box 1 (Drosophila)
INO80D	0.74	0.80	INO80 complex subunit D
KLF15	0.57	0.69	Kruppel-like factor 15
MARCH4	0.71	0.79	membrane-associated ring finger (C3HC4) 4
MSRA	0.79	0.75	methionine sulfoxide reductase A
PHLDB3	0.66	0.64	pleckstrin homology-like domain, family B, member 3
SH2D3A	0.69	0.76	SH2 domain containing 3A
SLC9A1	0.78	0.83	solute carrier family 9 (sodium/hydrogen exchanger), member 1
TGFB1	0.79	0.84	transforming growth factor, beta 1
TSC22D4	0.70	0.83	TSC22 domain family, member 4
WASH1	0.33	0.52	similar to WAS protein family homolog 1

Supplementary Table 3 List of shRNA and siRNAs used in this study.

Gene	Sense sequence (5'-3')	Ref.
c-JUN_#1	UCCUGAAACAGAGCAUGACCCUAGA	1
c-JUN_#2	GAUGGAAACGACCUUCUAUUU	2
CXCL1_#1	GCACAUCUGUUUUGUAACU	3
CXCL1_#2	CAGUGUUUCUGGCUUAGAA	4
FGFR1_#1	GCCACACUCUGCACCGCUA	5
FGFR1_#2	CCACAGAAUUGGAGGCUAC	6
FGFR3_#1	CGCCAAGCCUGUCACCGUA	7
FGFR3_#2	GACCGUGUCCUUACCGUGA	7
MmFgf2_#1	GUGUUUCUUCUUUGAACGACU	8
MmFgf2_#2	GCACUGAAACGAACUGGGCAGUAU	9
KRAS	SMARTpool: siGENOME (M-005069-00)	
GALNT14	Mission shRNA TRC Number: TRCN0000421183	

Supplementary Table 4 List of gene constructs used in this study.

Gene	Vector	Tag / Fusion protein	Source	Ref.
ActRIIB	pCS2	5'-c-Myc	Dr. Oelgeschläger	10
ALK3	pCS2	5'-HA	Dr. Oelgeschläger	10
c-JUN	pMIEG3	3'-IRES eGFP	Addgene (#40348)	11
FGFR1	pCMV	3'-EGFP	Dr. Heo	
FGFR3	pCMV	3'-mCit	Dr. Heo	
GALNT14	pBABE-hygro	5'-FLAG	Sinobiologicals	
SOX4	pBABE-hygro	5'-FLAG	Addgene (#36984)	12

Supplementary Table 5 List of qRT-PCR primers used in this study.

Gene		Sequence (5'-3')
c-JUN	Forward	GGCTACAGTAACCCCAAGA
	Reverse	GTGAGGAGGTCCGAGTTC
CXCL1	Forward	ACCCCAAGTTAGTTCAATCTG
	Reverse	GATCCGCCAGCCTCTATC
ETV1	Forward	AAGAGGAGAATTTGATTAGACTAAGT
	Reverse	CCATAAACAATAGCCACGGTATA
FGFR1	Forward	ATCTTGGCTTCTTACAGTTCTT
	Reverse	CTTAGTGAGGACAGTGATGAC
FGFR3	Forward	CCAAGCCTGTCACCGTAG
	Reverse	ATCATCTTCATCATCTCCATCTCA
GALNT1	Forward	TGATATGTAGAGCAGTCTGTTG
	Reverse	GCAGTGAAAGTCCTTGATAATC'
GALNT2	Forward	TTACTGCGGTTGCGTTAG
	Reverse	CGAAGGGCTCTTAGGAAAG
GALNT3	Forward	GCTCAAGGTCTCGTTCAG
	Reverse	TCCCATATCTGCTCTCCA
GALNT4	Forward	AGCAGCCTTAAAGTTTATCAAA
	Reverse	GAGAAGTGGGTGGGTTTAG
GALNT5	Forward	AATGTGGAGAATGGTGTATAGC
	Reverse	CTGGATGAAAGACTGATGTTGA
GALNT6	Forward	CCTTGCTATTATTACCTGAAGTCT
	Reverse	GTTTCTGTCCTGTCTTTCTCA
GALNT7	Forward	GCTGCTGCCATAATGACT
	Reverse	AGTGATATTACCAACTTACAAAGC
GALNT9	Forward	AGCCTTCTTGCCTGACTC
	Reverse	CCACATCCTCACACTTCTTC
GALNT10	Forward	GCACTCTGGCATTTGTCAT
	Reverse	AGTCAAGTTCTGTCCTTCCT
GALNT11	Forward	GGTGAAGAAGTGAGTGTCC
	Reverse	GATACTGTGGTTTGCCTCTAT
GALNT12	Forward	ATGTTATGAAGCCTCGTGTATC
	Reverse	TTTGGTGGGTTCTGGTCA
GALNT13	Forward	TCTGTTTCAACTCTCGTCTAATC
	Reverse	GTGGATATGGTGGCAAGTC
GALNT14 (3'UTR)	Forward	TCATTCCCTGATTGGTATCTG
	Reverse	AGAGACTGCTTCCTTTGTAG
GALNT14 (ORF)	Forward	TGTCAGTCATCACCTTGTTC
	Reverse	CATTGCTGTCGGTCATCT
GALNT16	Forward	AACCTCTGCTCTGGATTGAA

	Reverse	AGACACTTGGCTGCTGAC
GALNT18	Forward	CCAGAACCGCAAGTCTAAGC
	Reverse	CACTAACCTGGTTCCCCAGA
GALNT19	Forward	CCTCTGGTTAGGGTGCACAT
	Reverse	GCTAGGTCAGCATCGTCACA
GAPDH	Forward	CATGGGGAAGGTGAAGGTCG
	Reverse	GTTGAGGTCAATGAAGGGGT
HES1	Forward	CACAGAAAGTCATCAAAGCCTAT
	Reverse	TCCAGAATGTCCGCCTTC
HPRT	Forward	TGACCTTGATTTATTTTGCATACC
	Reverse	CGAGCAAGACGTTCAGTCCT
NANOG	Forward	GGCTGAATCCTTCCTCTC
	Reverse	GCTCCAACCATACTCCAC
OCT4	Forward	AACTGGTGTGTTTATGTTCTTAC
	Reverse	CCTCTTCTGCTTCAGGAG
SOX2	Forward	TAGTCTCCAAGCGACGAA
	Reverse	AGCAAGAAGCCTCTCCTT
SOX4	Forward	ACTCCTCCTCTTCCTCCT
	Reverse	CAGGGACATGCTCTCAAAG
SPRY1	Forward	TTCCCTGTTTCCCACCTT
	Reverse	GCTTCCCAGTCCACTCTT
MmFgf2	Forward	GAGAAGAGCGACCCACAC
	Reverse	ACACACTTAGAAGCCAGCAG
MmHPRT	Forward	AGCCAAATACAAAGCCTAAGAT
	Reverse	ACAGTAACATTCATAGAAGGTTCA
ANGPTL4	Assay ID	Hs01101127
B2M	Assay ID	Hs00984230
GALNT8	Assay ID	Hs00213610
GALNT15	Assay ID	Hs00365065
GALNT17	Assay ID	Hs01391759
GALNT20	Assay ID	Hs00377152
MmB2M	Assay ID	Mm00437762
MmGALNT14	Assay ID	Mm01233030

Supplementary Table 6 List of antibodies used in this study.

Antibody	Company	Catalog no.	Source	kDa	Dilution
AKT	Cell signaling	4691	Rabbit	60	1:2,000
pAKT (S473)	Cell signaling	4060	Rabbit	60	1:1,000
BMPR-1A	Santa Cruz Biotechnology	20736	Rabbit	66	1:3,000
β-Catenin	Cell signaling	8480	Rabbit	92	1:1,000
Claudin-1	Cell signaling	13255	Rabbit	20	1:1,000
c-JUN	Santa Cruz Biotechnology	44	Rabbit	39	1:1,000
p-c-JUN (S63/73)	Santa Cruz Biotechnology	16312	Rabbit	39	1:1,000
c-Myc-Tag	Abcam	9106	Rabbit		1:5,000
ERK1/2	Cell signaling	4695	Rabbit	42, 44	1:2,000
pERK1/2 (T202/Y204)	Cell signaling	9101	Rabbit	42, 44	1:2,000
FGFR1	Cell signaling	9740	Rabbit	92, 120, 145	1:1,000
FGFR3	Cell signaling	4574	Rabbit	125, 145, 165	1:1,000
GALNT14	GeneTex	122371	Rabbit	64	1:5,000
HA-Tag	Cell signaling	3724	Rabbit		1:1,000
KRAS	Santa Cruz Biotechnology	30	Mouse	21	1:1,000
Lamin B	Santa Cruz Biotechnology	6217	Goat	67	1:1,000
N-Cadherin	Cell signaling	13116	Rabbit	140	1:1,000
Slug	Cell signaling	9585	Rabbit	30	1:1,000
SMAD1	Cell signaling	6944	Rabbit	60	1:1,000
SMAD2	Cell signaling	5339	Rabbit	60	1:1,000
SMAD3	Cell signaling	9523	Rabbit	52	1:1,000
SMAD4	Cell signaling	9515	Rabbit	70	1:1,000
SMAD4 (also used for IP)	Santa Cruz Biotechnology	7966	Mouse	70	1:1,000
SMAD5	Cell signaling	12534	Rabbit	60	1:1,000
pSMAD2 (S465/467)	Cell signaling	3108	Rabbit	60	1:1,000
pSMAD3 (S423/425)	Cell signaling	9520	Rabbit	52	1:1,000
pSMAD1/5 (S463/465) /8 (S426/428)	Cell signaling	9511	Rabbit	60	1:1,000
pSMAD1/5 (S463/465) /9 (S465/467)	Cell signaling	13820	Rabbit	60	1:1,000
Tubulin	Millipore	05-829	Mouse	55	1:10,000

Vimentin	Cell signaling	5741	Rabbit	57	1:1,000
ZEB1	Cell signaling	3396	Rabbit	200	1:1,000
ZO-1	Cell signaling	8193	Rabbit	220	1:1,000

Supplementary References

1 Yu, B. *et al.* c-Jun protects hypoxia-inducible factor-1alpha from degradation via its oxygen-dependent degradation domain in a nontranscriptional manner. *Cancer research* **69**, 7704-7712, doi:10.1158/0008-5472.CAN-09-0808 (2009).

2 Naderi, A., Liu, J. & Francis, G. D. A feedback loop between BEX2 and ErbB2 mediated by c-Jun signaling in breast cancer. *International journal of cancer. Journal international du cancer* **130**, 71-82, doi:10.1002/ijc.25977 (2012).

3 Xu, J. *et al.* Lymphatic endothelial cell-secreted CXCL1 stimulates lymphangiogenesis and metastasis of gastric cancer. *International journal of cancer. Journal international du cancer* **130**, 787-797, doi:10.1002/ijc.26035 (2012).

4 Bolitho, C., Hahn, M. A., Baxter, R. C. & Marsh, D. J. The chemokine CXCL1 induces proliferation in epithelial ovarian cancer cells by transactivation of the epidermal growth factor receptor. *Endocrine-related cancer* **17**, 929-940, doi:10.1677/ERC-10-0107 (2010).

5 Cheung, L. W., Leung, K. W., Wong, C. K., Wong, R. N. & Wong, A. S. Ginsenoside-Rg1 induces angiogenesis via non-genomic crosstalk of glucocorticoid receptor and fibroblast growth factor receptor-1. *Cardiovascular research* **89**, 419-425, doi:10.1093/cvr/cvq300 (2011).

6 Cheng, C. L. *et al.* Expression of FGFR1 is an independent prognostic factor in triple-negative breast cancer. *Breast cancer research and treatment* **151**, 99-111, doi:10.1007/s10549-015-3371-x (2015).

7 Fawdar, S. *et al.* Targeted genetic dependency screen facilitates identification of actionable mutations in FGFR4, MAP3K9, and PAK5 in lung cancer. *Proceedings of the National Academy of Sciences of the United States of America* **110**, 12426-12431, doi:10.1073/pnas.1305207110 (2013).

8 Yang, J. *et al.* Binding of FGF2 to FGFR2 in an autocrine mode in trophectoderm cells is indispensable for mouse blastocyst formation through PKC-p38 pathway. *Cell cycle* **14**, 3318-3330, doi:10.1080/15384101.2015.1087622 (2015).

9 Izikki, M. *et al.* Endothelial-derived FGF2 contributes to the progression of pulmonary hypertension in humans and rodents. *The Journal of clinical investigation* **119**, 512-523, doi:10.1172/JCI35070 (2009).

10 Herr, P., Korniychuk, G., Yamamoto, Y., Grubisic, K. & Oelgeschlager, M. Regulation of TGF-(beta) signalling by N-acetylgalactosaminyltransferase-like 1. *Development* **135**, 1813-1822, doi:10.1242/dev.019323 (2008).

11 Wang, Z. Y. *et al.* Regulation of IL-10 gene expression in Th2 cells by Jun proteins. *Journal of immunology* **174**, 2098-2105 (2005).

12 Guo, W. *et al.* Slug and Sox9 cooperatively determine the mammary stem cell state. *Cell* **148**, 1015-1028, doi:10.1016/j.cell.2012.02.008 (2012).
